# Genetic Overlap Between Alzheimer’s Disease and Bipolar Disorder Implicates the MARK2 and VAC14 Genes

**DOI:** 10.3389/fnins.2019.00220

**Published:** 2019-03-13

**Authors:** Ole Kristian Drange, Olav Bjerkehagen Smeland, Alexey A. Shadrin, Per Ivar Finseth, Aree Witoelar, Oleksandr Frei, Psychiatric Genomics Consortium Bipolar Disorder Working Group, Eli A Stahl, Yunpeng Wang, Sahar Hassani, Srdjan Djurovic, Anders M. Dale, Ole A. Andreassen

**Affiliations:** ^1^ Department of Genetics and Genomic Sciences, Icahn School of Medicine at Mount Sinai, New York, NY, US; ^2^ Department of Psychiatry, Icahn School of Medicine at Mount Sinai, New York, NY, US; ^3^ Medical and Population Genetics, Broad Institute, Cambridge, MA, US; ^4^ MRC Social, Genetic and Developmental Psychiatry Centre, King’s College London, London, GB; ^5^ NIHR BRC for Mental Health, King’s College London, London, GB; ^6^ Department of Biomedicine, University of Basel, Basel, CH; ^7^ Department of Psychiatry (UPK), University of Basel, Basel, CH; ^8^ Institute of Human Genetics, University of Bonn, Bonn, DE; ^9^ Life&Brain Center, Department of Genomics, University of Bonn, Bonn, DE; ^10^ Institute of Medical Genetics and Pathology, University Hospital Basel, Basel, CH; ^11^ Division of Psychiatry, University College London, London, GB; ^12^ Stanley Center for Psychiatric Research, Broad Institute, Cambridge, MA, US; ^13^ Department of Psychiatry and Psychotherapy, Chariteì - Universitätsmedizin, Berlin, DE; ^14^ Analytic and Translational Genetics Unit, Massachusetts General Hospital, Boston, MA, US; ^15^ iSEQ, Center for Integrative Sequencing, Aarhus University, Aarhus, DK; ^16^ Department of Biomedicine - Human Genetics, Aarhus University, Aarhus, DK; ^17^ Department of Clinical Neuroscience, Centre for Psychiatry Research, Karolinska Institutet, Stockholm, SE; ^18^ Department of Psychiatry, Psychosomatics and Psychotherapy, Center of Mental Health, University Hospital Würzburg, Würzburg, DE; ^19^ iPSYCH, The Lundbeck Foundation Initiative for Integrative Psychiatric Research, DK; ^20^ Institute of Biological Psychiatry, Mental Health Centre Sct. Hans, Copenhagen, DK; ^21^ Institute of Clinical Medicine, University of Oslo, Oslo, NO; ^22^ Department of Complex Trait Genetics, Center for Neurogenomics and Cognitive Research, Amsterdam Neuroscience, Vrije Universiteit Amsterdam, Amsterdam, NL; ^23^ deCODE Genetics / Amgen, Reykjavik, IS; ^24^ Queensland Brain Institute, The University of Queensland, Brisbane, QLD, AU; ^25^ Institute for Molecular Bioscience, The University of Queensland, Brisbane, QLD, AU; ^26^ Division of Endocrinology and Center for Basic and Translational Obesity Research, Boston Children’s Hospital, Boston, MA, US; ^27^ Medical Research Council Centre for Neuropsychiatric Genetics and Genomics, Division of Psychological Medicine and Clinical Neurosciences, Cardiff University, Cardiff, GB; ^28^ National Centre for Register-Based Research, Aarhus University, Aarhus, DK; ^29^ Centre for Integrated Register-based Research, Aarhus University, Aarhus, DK; ^30^ Molecular & Behavioral Neuroscience Institute, University of Michigan, Ann Arbor, MI, US; ^31^ NEUROSCIENCE, Istituto Di Ricerche Farmacologiche Mario Negri, Milano, IT; ^32^ Department of Psychiatry and Behavioral Neuroscience, University of Chicago, Chicago, IL, US; ^33^ Psychiatry, Berkshire Healthcare NHS Foundation Trust, Bracknell, GB; ^34^ Psychiatry, Rush University Medical Center, Chicago, IL, US; ^35^ Center for Neonatal Screening, Department for Congenital Disorders, Statens Serum Institut, Copenhagen, DK; ^36^ Department of Psychiatry, Weill Cornell Medical College, New York, NY, US; ^37^ Department of Psychiatry and Psychotherapy, University Hospital Carl Gustav Carus, Technische Universität Dresden, Dresden, DE; ^38^ Department of Medical Epidemiology and Biostatistics, Karolinska Institutet, Stockholm, SE; ^39^ Department of Psychiatric Research, Diakonhjemmet Hospital, Oslo, NO; ^40^ Psychiatry, UMC Utrecht Hersencentrum Rudolf Magnus, Utrecht, NL; ^41^ Human Genetics, University of California Los Angeles, Los Angeles, CA, US; ^42^ Institute of Psychiatric Phenomics and Genomics (IPPG), University Hospital, LMU Munich, Munich, DE; ^43^ Department of Psychiatry and Human Behavior, University of California, Irvine, Irvine, CA, US; ^44^ Molecular & Behavioral Neuroscience Institute and Department of Computational Medicine & Bioinformatics, University of Michigan, Ann Arbor, MI, US; ^45^ Psychiatry, University of California San Francisco, San Francisco, CA, US; ^46^ Instituto de Salud Carlos III, Biomedical Network Research Centre on Mental Health (CIBERSAM), Madrid, ES; ^47^ Department of Psychiatry, Hospital Universitari Vall d ìHebron, Barcelona, ES; ^48^ Department of Psychiatry and Forensic Medicine, Universitat Autoìnoma de Barcelona, Barcelona, ES; ^49^ Psychiatric Genetics Unit, Group of Psychiatry Mental Health and Addictions, Vall d ìHebron Research Institut (VHIR), Universitat Autoìnoma de Barcelona, Barcelona, ES; ^50^ Department of Psychiatry, Mood Disorders Program, McGill University Health Center, Montreal, QC, CA; ^51^ Division of Psychiatry, University of Edinburgh, Edinburgh, GB; ^52^ University of Iowa Hospitals and Clinics, Iowa City, IA, US; ^53^ Translational Genomics, USC, Phoenix, AZ, US; ^54^ Department of Translational Research in Psychiatry, Max Planck Institute of Psychiatry, Munich, DE; ^55^ Centre for Psychiatry, Queen Mary University of London, London, GB; ^56^ UCL Genetics Institute, University College London, London, GB; ^57^ Department of Psychiatry, Laboratory of Psychiatric Genetics, Poznan University of Medical Sciences, Poznan, PL; ^58^ Department of Neurosciences, University of California San Diego, La Jolla, CA, US; ^59^ Department of Radiology, University of California San Diego, La Jolla, CA, US; ^60^ Department of Psychiatry, University of California San Diego, La Jolla, CA, US; ^61^ Department of Cognitive Science, University of California San Diego, La Jolla, CA, US; ^62^ Applied Molecular Genomics Unit, VIB Department of Molecular Genetics, University of Antwerp, Antwerp, Belgium; ^63^ Department of Psychiatry and Behavioral Sciences, Johns Hopkins University School of Medicine, Baltimore, MD, US; ^64^ Department of Medical Genetics, Oslo University Hospital Ullevål, Oslo, NO; ^65^ NORMENT, KG Jebsen Centre for Psychosis Research, Department of Clinical Science, University of Bergen, Bergen, NO; ^66^ Department of Neurology, Oslo University Hospital, Oslo, NO; ^67^ NORMENT, KG Jebsen Centre for Psychosis Research, Oslo University Hospital, Oslo, NO; ^68^ Center for Statistical Genetics and Department of Biostatistics, University of Michigan, Ann Arbor, MI, US; ^69^ Department of Medical & Molecular Genetics, Indiana University, Indianapolis, IN, US; ^70^ Department of Genetic Epidemiology in Psychiatry, Central Institute of Mental Health, Medical Faculty Mannheim, Heidelberg University, Mannheim, DE; ^71^ Center for Neurobehavioral Genetics, University of California Los Angeles, Los Angeles, CA, US; ^72^ Department of Molecular Medicine and Surgery, Karolinska Institutet and Center for Molecular Medicine, Karolinska University Hospital, Stockholm, SE; ^73^ Department of Clinical Neuroscience, Karolinska Institutet and Center for Molecular Medicine, Karolinska University Hospital, Stockholm, SE; ^74^ Child and Adolescent Psychiatry Research Center, Stockholm, SE; ^75^ Department of Psychiatry and Psychotherapy, University Medical Center GÖttingen, GÖttingen, DE; ^76^ Department of Psychiatry, Dalhousie University, Halifax, NS, CA; ^77^ Genetics and Computational Biology, QIMR Berghofer Medical Research Institute, Brisbane, QLD, AU; ^78^ Department of Psychological Medicine, University of Worcester, Worcester, GB; ^79^ School of Biomedical and Healthcare Sciences, Plymouth University Peninsula Schools of Medicine and Dentistry, Plymouth, GB; ^80^ School of Psychiatry, University of New South Wales, Sydney, NSW, AU; ^81^ Bioinformatics Research Centre, Aarhus University, Aarhus, DK; ^82^ Biostatistics, University of Minnesota System, Minneapolis, MN, US; ^83^ Mental Health Department, University Regional Hospital, Biomedicine Institute (IBIMA), Maìlaga, ES; ^84^ Department of Psychology, Eberhard Karls Universität Tübingen, Tubingen, DE; ^85^ Department of Psychiatry and Behavioral Sciences, Howard University Hospital, Washington, DC, US; ^86^ Center for Multimodal Imaging and Genetics, University of California San Diego, La Jolla, CA, US; ^87^ Psychiatrie Translationnelle, Inserm U955, Creìteil, FR; ^88^ Faculteì de Meìdecine, Universiteì Paris Est, Creìteil, FR; ^89^ Campbell Family Mental Health Research Institute, Centre for Addiction and Mental Health, Toronto, ON, CA; ^90^ Neurogenetics Section, Centre for Addiction and Mental Health, Toronto, ON, CA; ^91^ Department of Psychiatry, University of Toronto, Toronto, ON, CA; ^92^ Institute of Medical Sciences, University of Toronto, Toronto, ON, CA; ^93^ Department of Psychiatry, Psychosomatic Medicine and Psychotherapy, University Hospital Frankfurt, Frankfurt am Main, DE; ^94^ Cell Biology, SUNY Downstate Medical Center College of Medicine, Brooklyn, NY, US; ^95^ Institute for Genomic Health, SUNY Downstate Medical Center College of Medicine, Brooklyn, NY, US; ^96^ Center for Research in Environmental Epidemiology (CREAL), Barcelona, ES; ^97^ Psychiatry, Altrecht, Utrecht, NL; ^98^ Psychiatry, GGZ inGeest, Amsterdam, NL; ^99^ Psychiatry, VU medisch centrum, Amsterdam, NL; ^100^ Psychiatry, North East London NHS Foundation Trust, Ilford, GB; ^101^ Clinic for Psychiatry and Psychotherapy, University Hospital Cologne, Cologne, DE; ^102^ Psychiatric and Neurodevelopmental Genetics Unit, Massachusetts General Hospital, Boston, MA, US; ^103^ HudsonAlpha Institute for Biotechnology, Huntsville, AL, US; ^104^ Department of Human Genetics, University of Michigan, Ann Arbor, MI, US; ^105^ Psychiatry, University of Illinois at Chicago College of Medicine, Chicago, IL, US; ^106^ Max Planck Institute of Psychiatry, Munich, DE; ^107^ Mental Health, NHS 24, Glasgow, GB; ^108^ Division of Psychiatry, Centre for Clinical Brain Sciences, University of Edinburgh, Edinburgh, GB; ^109^ Psychiatry, Brigham and Women’s Hospital, Boston, MA, US; ^110^ Department of Psychiatry and Psychotherapy, University of Bonn, Bonn, DE; ^111^ Department of Genetics, Harvard Medical School, Boston, MA, US; ^112^ Department of Psychiatry, University of Michigan, Ann Arbor, MI, US; ^113^ Genetic Cancer Susceptibility Group, International Agency for Research on Cancer, Lyon, FR; ^114^ Estonian Genome Center, University of Tartu, Tartu, EE; ^115^ Discipline of Biochemistry, Neuroimaging and Cognitive Genomics (NICOG) Centre, National University of Ireland, Galway, Galway, IE; ^116^ Neuropsychiatric Genetics Research Group, Dept of Psychiatry and Trinity Translational Medicine Institute, Trinity College Dublin, Dublin, IE; ^117^ Institute of Neuroscience and Medicine (INM-1), Research Centre Jülich, Jülich, DE; ^118^ Research/Psychiatry, Veterans Affairs San Diego Healthcare System, San Diego, CA, US; ^119^ Department of Clinical Sciences, Psychiatry, Umeå University Medical Faculty, Umeå, SE; ^120^ Department of Clinical Psychiatry, Psychiatry Clinic, Clinical Center University of Sarajevo, Sarajevo, BA; ^121^ Department of Neurobiology, Care sciences, and Society, Karolinska Institutet and Center for Molecular Medicine, Karolinska University Hospital, Stockholm, SE; ^122^ Psychiatry, Harvard Medical School, Boston, MA, US; ^123^ Division of Clinical Research, Massachusetts General Hospital, Boston, MA, US; ^124^ Outpatient Clinic for Bipolar Disorder, Altrecht, Utrecht, NL; ^125^ Department of Psychiatry, Washington University in Saint Louis, Saint Louis, MO, US; ^126^ Department of Biochemistry and Molecular Biology II, Institute of Neurosciences, Center for Biomedical Research, University of Granada, Granada, ES; ^127^ Department of Neuroscience, Icahn School of Medicine at Mount Sinai, New York, NY, US; ^128^ Medicine, Psychiatry, Biomedical Informatics, Vanderbilt University Medical Center, Nashville, TN, US; ^129^ Department of Health Sciences Research, Mayo Clinic, Rochester, MN, US; ^130^ Psychiatry and Behavioral Sciences, Stanford University School of Medicine, Stanford, CA, US; ^131^ Rush University Medical Center, Chicago, IL, US; ^132^ Scripps Translational Science Institute, La Jolla, CA, US; ^133^ Neuroscience Research Australia, Sydney, NSW, AU; ^134^ Faculty of Medicine, Department of Psychiatry, School of Health Sciences, University of Iceland, Reykjavik, IS; ^135^ Div Mental Health and Addiction, Oslo University Hospital, Oslo, NO; ^136^ NORMENT, University of Oslo, Oslo, NO; ^137^ Psychiatry and the Behavioral Sciences, University of Southern California, Los Angeles, CA, US; ^138^ Mood Disorders, PsyQ, Rotterdam, NL; ^139^ Institute for Medical Sciences, University of Aberdeen, Aberdeen, UK; ^140^ Research Division, Federal Institute for Drugs and Medical Devices (BfArM), Bonn, DE; ^141^ Centre for Addiction and Mental Health, Toronto, ON, CA; ^142^ Neurogenomics, TGen, Los Angeles, AZ, US; ^143^ Psychiatry, Psychiatrisches Zentrum Nordbaden, Wiesloch, DE; ^144^ Computational Sciences Center of Emphasis, Pfizer Global Research and Development, Cambridge, MA, US; ^145^ Department of Biostatistics, Princess Margaret Cancer Centre, Toronto, ON, CA; ^146^ Dalla Lana School of Public Health, University of Toronto, Toronto, ON, CA; ^147^ Psychological Medicine, Institute of Psychiatry, Psychology & Neuroscience, King’s College London, London, GB; ^148^ Department of Mental Health, Johns Hopkins University Bloomberg School of Public Health, Baltimore, MD, US; ^149^ Institute of Genetic Medicine, Johns Hopkins University School of Medicine, Baltimore, MD, US; ^150^ NORMENT, KG Jebsen Centre for Psychosis Research, Division of Mental Health and Addiction, Institute of Clinical Medicine and Diakonhjemmet Hospital, University of Oslo, Oslo, NO; ^151^ National Institute of Mental Health, Klecany, CZ; ^152^ Discipline of Psychiatry, University of Adelaide, Adelaide, SA, AU; ^153^ Department of Psychiatry and Addiction Medicine, Assistance Publique - Ho^pitaux de Paris, Paris, FR; ^154^ Paris Bipolar and TRD Expert Centres, FondaMental Foundation, Paris, FR; ^155^ UMR-S1144 Team 1: Biomarkers of relapse and therapeutic response in addiction and mood disorders, INSERM, Paris, FR; ^156^ Psychiatry, Universiteì Paris Diderot, Paris, FR; ^157^ Psychiatry, University of Pennsylvania, Philadelphia, PA, US; ^158^ Department of Psychiatry, University of Münster, Münster, DE; ^159^ Division of Endocrinology, Children’s Hospital Boston, Boston, MA, US; ^160^ Centre for Affective Disorders, Institute of Psychiatry, Psychology and Neuroscience, London, GB; ^161^ Department of Psychiatry & Psychology, Mayo Clinic, Rochester, MN, US; ^162^ School of Medical Sciences, University of New South Wales, Sydney, NSW, AU; ^163^ Department of Human Genetics, University of Chicago, Chicago, IL, US; ^164^ Biometric Psychiatric Genetics Research Unit, Alexandru Obregia Clinical Psychiatric Hospital, Bucharest, RO; ^165^ Institute of Neuroscience and Physiology, University of Gothenburg, Gothenburg, SE; ^166^ INSERM, Paris, FR; ^167^ Department of Medical & Molecular Genetics, King’s College London, London, GB; ^168^ Neuroscience Therapeutic Area, Janssen Research and Development, LLC, Titusville, NJ, US; ^169^ Cancer Epidemiology and Prevention, M. Sklodowska-Curie Cancer Center and Institute of Oncology, Warsaw, PL; ^170^ School of Psychology, The University of Queensland, Brisbane, QLD, AU; ^171^ Research Institute, Lindner Center of HOPE, Mason, OH, US; ^172^ Centre for Cognitive Ageing and Cognitive Epidemiology, University of Edinburgh, Edinburgh, GB; ^173^ Human Genetics Branch, Intramural Research Program, National Institute of Mental Health, Bethesda, MD, US; ^174^ Division of Mental Health and Addiction, Oslo University Hospital, Oslo, NO; ^175^ Division of Mental Health and Addiction, University of Oslo, Institute of Clinical Medicine, Oslo, NO; ^176^ Institute of Molecular and Cell Biology, University of Tartu, Tartu, EE; ^177^ Mental Health, Faculty of Medicine and Health Sciences, Norwegian University of Science and Technology - NTNU, Trondheim, NO; ^178^ Psychiatry, St Olavs University Hospital, Trondheim, NO; ^179^ Psychosis Research Unit, Aarhus University Hospital, Risskov, DK; ^180^ Munich Cluster for Systems Neurology (SyNergy), Munich, DE; ^181^ University of Liverpool, Liverpool, GB; ^182^ Psychiatry and Human Genetics, University of Pittsburgh, Pittsburgh, PA, US; ^183^ Mental Health Services in the Capital Region of Denmark, Mental Health Center Copenhagen, University of Copenhagen, Copenhagen, DK; ^184^ Division of Psychiatry, Haukeland Universitetssjukehus, Bergen, NO; ^185^ Faculty of Medicine and Dentistry, University of Bergen, Bergen, NO; ^186^ Human Genetics and Computational Biomedicine, Pfizer Global Research and Development, Groton, CT, US; ^187^ College of Medicine Institute for Genomic Health, SUNY Downstate Medical Center College of Medicine, Brooklyn, NY, US; ^188^ Department of Clinical Genetics, Amsterdam Neuroscience, Vrije Universiteit Medical Center, Amsterdam, NL; ^189^ Department of Neurology and Neurosurgery, McGill University, Faculty of Medicine, Montreal, QC, CA; ^190^ Montreal Neurological Institute and Hospital, Montreal, QC, CA; ^191^ Department of Biomedical and NeuroMotor Sciences, University of Bologna, Bologna, IT; ^192^ Department of Psychiatry, Massachusetts General Hospital, Boston, MA, US; ^193^ Psychiatric and Neurodevelopmental Genetics Unit (PNGU), Massachusetts General Hospital, Boston, MA, US; ^194^ Faculty of Medicine, University of Iceland, Reykjavik, IS; ^195^ Department of Psychiatry, Hospital Namsos, Namsos, NO; ^196^ Department of Neuroscience, Norges Teknisk Naturvitenskapelige Universitet Fakultet for naturvitenskap og teknologi, Trondheim, NO; ^197^ Department of Genetics, University of North Carolina at Chapel Hill, Chapel Hill, NC, US; ^198^ Department of Psychiatry, University of North Carolina at Chapel Hill, Chapel Hill, NC, US; ^199^ Department of Psychiatry, McGill University, Montreal, QC, CA; ^200^ Dept of Psychiatry, Sankt Olavs Hospital Universitetssykehuset i Trondheim, Trondheim, NO; ^201^ Clinical Institute of Neuroscience, Hospital Clinic, University of Barcelona, IDIBAPS, CIBERSAM, Barcelona, ES; ^202^ Institute of Biological Psychiatry, MHC Sct. Hans, Mental Health Services Copenhagen, Roskilde, DK; ^203^ Department of Clinical Medicine, University of Copenhagen, Copenhagen, DK; ^204^ Psychiatry, Indiana University School of Medicine, Indianapolis, IN, US; ^205^ Biochemistry and Molecular Biology, Indiana University School of Medicine, Indianapolis, IN, US; ^†^ Equal contribution; ^*^ Co-last authors; ^^^ deceased; ^1^Department of Research and Development, Department of Mental Health, Norwegian University of Science and Technology, Trondheim, Norway; ^2^Department of Østmarka, Division of Mental Health Care, St. Olavs Hospital, Trondheim University Hospital, Trondheim, Norway; ^3^Norwegian Centre for Mental Disorders Research, KG Jebsen Centre for Psychosis Research, Institute of Clinical Medicine, University of Oslo, Oslo, Norway; ^4^Norwegian Centre for Mental Disorders Research, Division of Mental Health and Addiction, Oslo University Hospital, Oslo, Norway; ^5^Department of Brøset, Division of Mental Health Care, St. Olavs Hospital, Trondheim University Hospital, Trondheim, Norway; ^6^Department of Medical Genetics, Oslo University Hospital, Oslo, Norway; ^7^Norwegian Centre for Mental Disorders Research, KG Jebsen Centre for Psychosis Research, Department of Clinical Science, University of Bergen, Bergen, Norway; ^8^Center for Multimodal Imaging and Genetics, Department of Radiology, University of California, San Diego, La Jolla, CA, United States; ^9^Department of Neurosciences, University of California, San Diego, La Jolla, CA, United States; ^10^Department of Psychiatry, University of California, San Diego, La Jolla, CA, United States

**Keywords:** Alzheimer’s disease, bipolar disorder, GWAS, pleiotropy, cognitive symptoms, affective symptoms, MARK2, VAC14

## Abstract

**Background:** Alzheimer’s disease (AD) and bipolar disorder (BIP) are complex traits influenced by numerous common genetic variants, most of which remain to be detected. Clinical and epidemiological evidence suggest that AD and BIP are related. However, it is not established if this relation is of genetic origin. Here, we applied statistical methods based on the conditional false discovery rate (FDR) framework to detect genetic overlap between AD and BIP and utilized this overlap to increase the power to identify common genetic variants associated with either or both traits.

**Methods:** We obtained genome wide association studies data from the International Genomics of Alzheimer’s Project part 1 (17,008 AD cases and 37,154 controls) and the Psychiatric Genetic Consortium Bipolar Disorder Working Group (20,352 BIP cases and 31,358 controls). We used conditional QQ-plots to assess overlap in common genetic variants between AD and BIP. We exploited the genetic overlap to re-rank test-statistics for AD and BIP and improve detection of genetic variants using the conditional FDR framework.

**Results:** Conditional QQ-plots demonstrated a polygenic overlap between AD and BIP. Using conditional FDR, we identified one novel genomic locus associated with AD, and nine novel loci associated with BIP. Further, we identified two novel loci jointly associated with AD and BIP implicating the *MARK2* gene (lead SNP rs10792421, conjunctional FDR = 0.030, same direction of effect) and the *VAC14* gene (lead SNP rs11649476, conjunctional FDR = 0.022, opposite direction of effect).

**Conclusion:** We found polygenic overlap between AD and BIP and identified novel loci for each trait and two jointly associated loci. Further studies should examine if the shared loci implicating the *MARK2* and *VAC14* genes could explain parts of the shared and distinct features of AD and BIP.

## Introduction

About a century ago, Alois Alzheimer and Emil Kraepelin described the historical equivalents of AD and BIP (Alzheimer, 1907; Kraepelin, 1921). Still their etiologies are incompletely understood and no curative treatments exist (Grande et al., 2016; Scheltens et al., 2016). The Global Burden of Disease study ranks AD and BIP among the top thirty causes of years lived with disability worldwide (Vos et al., 2016).

Alzheimer’s disease is a neurodegenerative disorder (Jack et al., 2013) usually presenting in late adult life (Koedam et al., 2010), while BIP is considered a neurodevelopmental disorder (Sanches et al., 2008; O’Shea and McInnis, 2016) with average age at onset in early adult life (Baldessarini et al., 2010). Yet, epidemiological, pathophysiological, and clinical data suggest that AD and BIP could be related. A recent meta-analysis reports an odds ratio of 2.4 (95% CI 1.4–4.1) for dementia of all causes among patients with BIP (Diniz et al., 2017). The risk of dementia is higher among patients with BIP compared to patients with arthritis, diabetes, and schizophrenia (Kessing et al., 1999; Kessing and Nilsson, 2003). Among patients with BIP, treatment with lithium is associated with a reduced risk of dementia (Kessing et al., 2010; Gerhard et al., 2015) and AD (Nunes et al., 2007) in most, but not all (Cheng et al., 2017), observational studies. Among patients with AD or mild cognitive impairment, a meta-analysis of randomized controlled studies found that lithium decreased cognitive decline (Matsunaga et al., 2015). Shared pathophysiological processes between AD and BIP are reported in the kynurenine pathway (Miller et al., 2006; Myint et al., 2007; Rahman et al., 2009; Gulaj et al., 2010; Maddison and Giorgini, 2015; Savitz et al., 2015). There is also evidence of inflammatory processes in both conditions (Goldstein et al., 2009; Antonio et al., 2015; Heneka et al., 2015). Further, euthymic patients with BIP have impairments of episodic memory (Torres et al., 2007) and executive dysfunction (Torres et al., 2007; Martino et al., 2015), which are also core symptoms of AD (Gold and Budson, 2008; Godefroy et al., 2016).

Despite several lines of evidence suggesting a relation between AD and BIP, it is not established if the conditions have a shared genetic basis. AD and BIP are in most cases complex traits, i.e., they are influenced by several genetic and environmental factors. Twin studies estimate the heritability of AD and BIP to 60% or higher (McGuffin et al., 2003; Kieseppä and Partonen, 2004; Gatz et al., 2006; Lichtenstein et al., 2009). Genome wide association studies (GWASs) are the gold standard for hypothesis-free assessment of associations between complex traits and common genetic variants (Corvin et al., 2010). The common variants refer to single nucleotide polymorphisms (SNPs) with minor allele frequencies > 1–5%. The power of a GWAS is a function of study sample size and the genetic architecture of the trait (i.e., the narrow-sense heritability, the number of causal variants, their effect sizes, and population frequencies) (Schork et al., 2016; Frei et al., 2018). AD and BIP are considered highly polygenic (Purcell et al., 2009; Escott-Price et al., 2015), and ∼1/3 of their heritability can be explained by SNPs with tiny effect sizes that are not individually detectable given the power of current GWASs (Lee et al., 2011, 2013; Ridge et al., 2013, 2016).

With the current sample sizes, however, the power of GWASs can be boosted by leveraging polygenic overlap between complex traits (Andreassen et al., 2013a,b, 2015). Shared genetic influences are common among complex traits (Visscher et al., 2017). Statistical methods based on the conditional FDR framework can detect polygenic overlap between complex traits and utilize this polygenic overlap to increase the power to identify common genetic variants associated with each trait and jointly with two or more traits (Andreassen et al., 2013a,b, 2015). We aimed to use these methods to identify the shared genetic basis between AD and BIP.

## Materials and Methods

### Data Sources

We obtained summary statistics (i.e., effect sizes and corresponding *p*-values for all SNPs) from the IGAP (Lambert et al., 2013) and the PGC2-BIP (Stahl et al., 2019).

#### International Genomics of Alzheimer’s Project

The IGAP is a two-stage study. We used data from stage 1 of the study, which is based upon four previously published GWASs [The European Alzheimer’s Disease Initiative (Dreses-Werringloer et al., 2008; Heath et al., 2008), the Alzheimer Disease Genetics Consortium (Jun et al., 2010), The Cohorts for Heart and Aging Research in Genomic Epidemiology consortium (Psaty et al., 2009), The Genetic and Environmental Risk in AD consortium (Harold et al., 2009)] on 17,008 AD cases and 37,154 controls of European ancestry. The IMPUTE2 (Howie et al., 2009) or MaCH/Minimac (Li et al., 2010) software were used to impute SNPs from the European ancestry haplotypes in the 1000 Genome Project (Altshuler et al., 2010). In stage 2 of the study, SNPs with *p*-values < 1 × 10^-3^ from stage 1 were selected for genotyping in independent samples. We did not use data from stage 2 of the study since the conditional FDR method require genome-wide summary statistics which are not inflated.

Diagnoses of AD in the sub-studies of IGAP were in most cases made clinically according to the National Institute of Neurological and Communicative Disorders and Stroke and the Alzheimer’s disease and Related Disorders Association criteria (McKhann et al., 1984) or the Diagnostic and Statistical Manual of Mental Disorders (American Psychiatric Association, 1994) criteria, or post mortem according to the National Institute of Ageing-Regan criteria (Newell et al., 1999).

Informed consents were provided from all participants, or, in the case of substantial cognitive impairment, from caregivers, legal guardians, or other proxies. The sub-studies were approved by local ethic committees.

For further details, we refer to the original publication (Lambert et al., 2013).

#### Psychiatric Genetic Consortium 2 Bipolar Disorder Working Group

The PGC2-BIP is a GWAS based upon 32 sub-studies on 20,352 BIP cases and 31,358 controls of European ancestry. Arrays for genotyping were chosen by each sub-study. The *Ricopoli* pipeline^1^ was used to standardize quality control, imputation, and analyses of genotypic data from all samples except one. SNPs were excluded by the following criteria: missingness in > 5 (before sample removal) or 2% (after sample removal), *p*-value for Hardy–Weinberg equilibrium < 1 × 10^-10^ in cases or <1 × 10^-6^ in controls, missingness difference between cases and controls > 2%, or autosomal heterozygosity deviation (|*F_het_*| > 0.2). Individuals with > 2% missing genotypes were also excluded. The IMPUTE2 (Howie et al., 2009) and SHAPEIT2 (Delaneau et al., 2012) software were used for imputation.

Diagnoses of BIP were established by clinical interviews or obtained from hospital record data according to the Diagnostic and Statistical Manual of Mental Disorders 4th edition (American Psychiatric Association, 1994), the International Classification of Diseases 9th revision (World Health Organization, 1977), or the International Classification of Diseases 10th revision (World Health Organization, 1992).

Informed consents were provided from all participants. The sub-studies were approved by local ethical committees.

For further details, we refer to the original publication (Stahl et al., 2019).

#### Data Availability

Data from the IGAP^2^ and PGC2-BIP^3^ studies are publicly available for download.

### Statistical Analyses

#### Conditional Quantile–Quantile (QQ)-Plots

We used conditional QQ-plots to visually assess pleiotropic enrichment. A conditional QQ-plot displays the distribution of p-values for the first trait, e.g., AD, conditioned on association levels for the second trait, e.g., BIP. Pleiotropic enrichment is present if the degree of leftward shift from the expected null line for the first trait is dependent on the degree of association with the second trait. For further details, we refer to previous studies (Andreassen et al., 2013a,b, 2015) and Supplementary Methods 1.1.

#### Conditional False Discovery Rate (condFDR)

The enrichment observed in conditional QQ-plots can be translated to FDR for each SNP. We used the conditional false discovery rate (condFDR) to improve power to detect SNPs associated with AD given associations with BIP and *vice versa*. condFDR is defined as “the posterior probability that a given SNP is null for the first trait given that the p-values for both traits are as small or smaller than the observed *p*-values” (Andreassen et al., 2015). We denoted condFDR for AD given associations with BIP as condFDR_(ADjBIP)_ and for BIP given association with AD as condFDR_(BIPj_ _AD)_ and considered values < 0.01 significant. For further details, we refer to previous studies (Andreassen et al., 2013a,b, 2015) and Supplementary Methods 1.2.

#### Conjunctional False Discovery Rate (conjFDR)

We used conjunctional FDR (conjFDR) to identify SNPs jointly associated with AD and BIP. conjFDR is defined as “the posterior probability that a SNP is null for either phenotype or both simultaneously, given the *p*-values for both traits are as small or smaller than the observed *p*-values” (Andreassen et al., 2015). After repeating the condFDR procedure for both traits, we identified shared loci at conjFDR < 0.05, which is given by the maximum between the condFDRs for both traits. Hence, the conjFDR analysis is a conservative approach requiring that loci exceed a condFDR significance threshold for two traits simultaneously. For further details, we refer to previous studies (Andreassen et al., 2013a,b, 2015) and Supplementary Methods 1.3.

#### Conditional and Conjunctional Manhattan Plots

We constructed conditional Manhattan plots to visualize the chromosomal location of SNPs with condFDR_(ADjBIP)_ (Supplementary Figure 1) and condFDR_(BIPjAD)_ < 0.01 (Supplementary Figure 2). We constructed a similar plot for SNPs jointly associated with AD and BIP at a conjFDR < 0.05 (Figure 2).

#### Assessment of Novelty

To determine if a locus was novel, we first checked that the p-value(s) for the implicating variant was > 5 × 10^-8^ in the original GWAS(s). Further, we used LDlink (Machiela and Chanock, 2015) to exclude variants which are in LD (*r*^2^> 0.1) with any of the genome-wide significant hits in the original GWAS(s). Finally, we conducted a search on PubMed using the term (“SNP id” OR “gene name”) AND (“Bipolar Disorder”[Mesh] OR “Alzheimer Disease”[Mesh]) to exclude that the variants or implicated genes have been associated with AD or BIP at genome-wide significance in previous GWASs.

#### Cerebral Gene Expression Across Lifespan of the Implicated Loci

The Human Brain Transcriptome (HBT) project^4^ used postmortem brain tissue from over 1,340 samples to provide genome-wide exon-level transcriptome data in 16 cerebral regions (Kang et al., 2011). We obtained figures from the HBT project on gene expression in different cerebral areas as a function of age (i.e., from embryonic life through late adulthood) for the nearest genes to the loci jointly associated with AD and BIP.

#### Control of Spurious Enrichment

We randomly chose one SNP in each LD block (*r*^2^> 0.1), and calculated the average empirical cumulative distribution function (ecdf) by using the *p*-values obtained through 200 iterations. SNPs within the major histocompatibility complex region (defined as chr6:25652429–33368333) and the apolipoprotein E (*APOE*) gene (chr19:44909039–45912650), and SNPs in LD (*r*^2^> 0.1) with these SNPs, were excluded from the analyses due to their complex LD structure (de Bakker and Raychaudhuri, 2012) and known association to AD (Lambert et al., 2010; Scheltens et al., 2016), which could bias the estimates of enrichment. Further, we used LD-independent (*r*^2^< 0.1) intergenic SNPs, which are depleted of true associations, to calculate an inflation factor value (Wang et al., 2016a). We divided all test statistics on this value to control for genomic inflation.

#### Cross-Trait Linkage Disequilibrium Score Regression (LDSR)

We calculated the degree of genetic correlation between AD and BIP using cross-trait LD score regression (LDSR) (Bulik-Sullivan et al., 2015). For details, we refer to Supplementary Materials 1.4.

### Ethics Statement

All GWASs performed and investigated in the present study were approved by the local ethics committees, and informed consent was obtained from all participants. Furthermore, the Norwegian Institutional Review Board for the South-East Norway Region has evaluated the methods used in the current study and found that no additional institutional review board approval was needed because no individual data were used (ref. 2011/1980).

## Results

### Pleiotropic Enrichment

**FIGURE 1:**
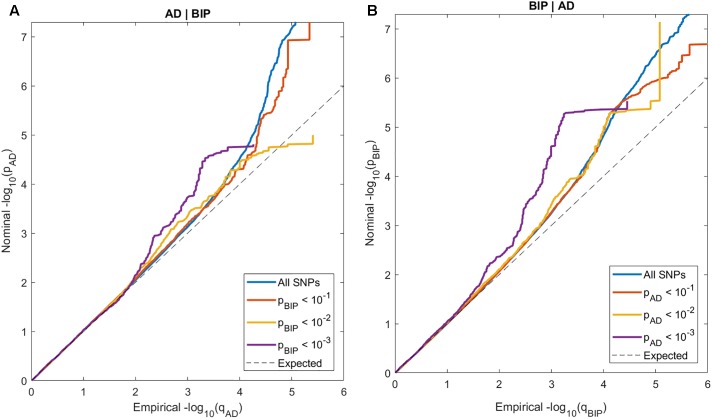
Conditional QQ-plots of nominal p-values at y-axis and 1 - empirical cumulative distribution function on *x*-axis for **(A)** Alzheimer’s disease (AD) with lines representing strata of SNPs according to their degree of association with bipolar disorder (BIP) and **(B)** BIP with lines representing strata of SNPs according to their degree of association with AD.

In the conditional QQ-plots, we observed enrichment of associations with AD given increasing SNP associations with BIP, and *vice versa* (Figure 1). These findings indicate polygenic overlap between AD and BIP across common genetic variants.

### Improving Genetic Discovery Using Conditional FDR

We then leveraged the pleiotropic enrichment observed in conditional QQ-plots to boost SNP discovery in both traits using condFDR.

We identified 22 SNPs clumped into 19 independent loci at condFDR_(ADjBIP)_ < 0.01 (Supplementary Table 1). The chromosomal locations of the nearest genes are visualized in a conditional Manhattan plot (Supplementary Figure 1). Red annotations represent the four loci with a lower conditional than unconditional FDR. Of these four loci, two loci have uncorrected *p*-values > 5 × 10^-8^ in the original GWAS and are thus not identified by traditional methods; *NDUFS3* (rs71475924, intron variant) and *MTSS1L* (rs12597717, intron variant). The signal in *NDUFS3* was driven by one single SNP and is thus probably a spurious association.

Further, we identified 24 SNPs within 24 loci at a condFDR_(BIPjAD)_ < 0.01 (Supplementary Table 2). As visualized in the conditional Manhatton plot (Supplementary Figure 2), 17 loci had a lower conditional than unconditional FDR. Of these 17 loci, 10 variants have uncorrected *p*-values > 5 × 10^-8^ in the original GWAS and are thus not identified by traditional methods; *LOC105378763* (rs1889778, intron variant), *CNTNAP5* (rs13011184, intron variant), *KIAA1109* (rs45605540, intron variant), *SSBP2* (rs7707981, intron variant), *AK091365* (rs2388334, no genic locational annotation), *RCOR2* (rs4980532, intron variant), *STARD9* (rs4447398, intron variant), *GRIN2A* (rs11647445, intron variant), *THRA* (rs61554907, intron variant), and *PRKCA* (rs7406066, intron variant). However; the *CNTNAP5* gene has previously been associated with the posterior cortical atrophy variant of AD at genome-wide significance (Schott et al., 2016) and with BIP (Djurovic et al., 2010).

### Identification of Shared Loci

Finally, we applied conjFDR to assess for SNPs jointly associated with AD and BIP. We used effect sizes from the original data sources to determine the allelic direction of effects in both traits.

**FIGURE 2:**
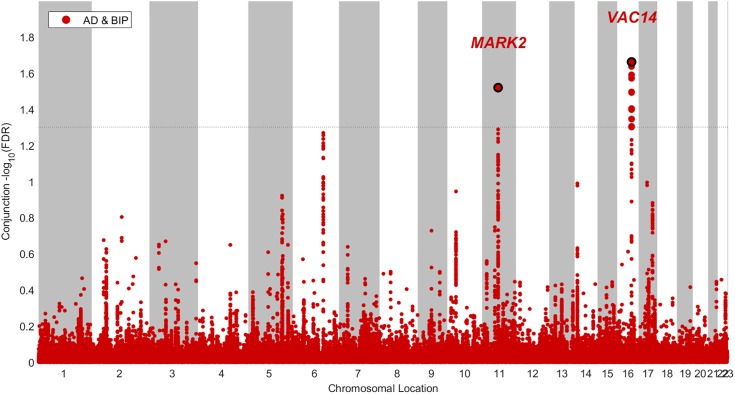
Conjunctional Manhatton plot of loci jointly associated with Alzheimer’s disease (AD) and bipolar disorder (BIP) at a conjuntional false discovery rate < 0.05.

We identified two SNPs at two loci at a conjFDR_(AD&BIP)_ < 0.05 (Table 1 and Figure 2). A 2 kb upstream variant at *MARK2* (rs10792421) was associated with AD and BIP with the same direction of effect on AD and BIP [conjFDR_(AD&BIP)_ = 0.030, *z*-score_(AD)_ = 3.99, *z*-score_(BIP)_ = 4.74]. *MARK2* is widely expressed in the developing and adult human brain (Supplementary Figure 3). An intronic variant within *VAC14* (rs11649476) was associated with AD and BIP with opposite directions of effect in AD and BIP [conjFDR_(AD&BIP)_ = 0.022, *z*-score_(AD)_ = -4.35, *z*-score_(BIP)_ = 4.18]. *VAC14* is also widely expressed in the developing and adult human brain (Supplementary Figure 4). Both SNPs have *p*-values > 5 × 10^-8^ for both traits in the original GWASs and are thus not identified by traditional methods.

**Table 1:** SNPs with related genes jointly associated with Alzheimer’s disease (AD) and bipolar disorder (BIP) at a conjunctional false discovery rate (conjFDR(_AD&BIP)_) < 0.05.

SNP	Chr. region	Position	Closest gene	Location relative to the closest gene	*P*-value_(AD)_	*P*-value_(BIP)_	conjFDR_(AD&BIP)_	Effective/ other allele	Direction of effect in AD/BIP
rs10792421	11q13.1	63605177	MARK2	Upstream	6.68E-05	2.16E-05	3.02E-02	G/A	+/+
rs11649476	16q22.2	70736752	VAC14	Intronic	1.35E-05	2.98E-05	2.18E-02	T/C	-/+


### Genetic Correlation

We estimated that there is no overall genetic correlation between AD and BIP according to LDSR (*r*_g_ = -0.0222, *SE* = 0.0519, *p* = 0.669).

## Discussion

We used statistical methods based on the condFDR framework and showed that AD and BIP have a shared genetic basis. Our study adds new insights into the relation between AD and BIP by finding polygenic overlap, one novel locus associated with AD and nine novel loci associated with BIP when conditioned on associations with the other trait, and two novel loci jointly associated with both traits.

A polygenic overlap between AD and BIP could implicate shared genetic influences as a part of the explanation to the epidemiological (Diniz et al., 2017), pathophysiological (Goldstein et al., 2009; Heneka et al., 2015), and clinical (Gold and Budson, 2008; Martino et al., 2015; Godefroy et al., 2016) links between the diseases. However, we do not find an overall genetic correlation as assessed with cross-trait LDSR (Bulik-Sullivan et al., 2015). Also, one of the two jointly associated SNPs demonstrates effects in opposite directions. These findings are compatible with a scenario where the polygenic overlap between AD and BIP involves a mixed direction of effects of the implicated SNPs yielding no genome-wide correlation (Frei et al., 2018). Thus, absence of an overall genetic correlation between brain disorders, as evident for several traits (including AD and BIP) in the study of Anttila et al. (2018), does not imply lack of genetic overlap.

The loci implicating the *MARK2* and *VAC14* genes were jointly associated with AD and BIP (Table 1). Both genes are widely expressed in the human brain throughout life (Supplementary Figures 3, 4), which implies a spatial and temporal relation to both neurodevelopmental and neurodegenerative processes. The locus implicating the *MARK2* gene (rs10792421) had a concordant direction of effect in both traits (Table 1). The *MARK2* gene encodes the microtubule affinity regulating kinase 2 (MARK2). The kinase is involved in a diversity of neuronal cellular processes, including neuronal migration, and tau phosphorylation (Matenia and Mandelkow, 2009). Migration of immature neurons is necessary for corticogenesis (Kon et al., 2017). BIP is considered a neurodevelopmental disorder partly because of previous findings of cortical cell migration abnormalities (Sanches et al., 2008; O’Shea and McInnis, 2016). Abnormal neuronal migration might also be involved in later stages of life among patients with AD (Reiner et al., 2009). Tauopathy is one of the pathophysiological hallmarks of AD (Jack et al., 2013). Gu G.J. et al. (2013) demonstrated that MARK2 increases the phosphorylation of tau *in situ* and found interactions between MARK2 and tau in postmortem human AD brain tissue. The role of tauopathy has also been explored in BIP. A study of cerebrospinal fluid among younger patients with BIP (Jakobsson et al., 2013) and a similar study of elderly patients with BIP and mild cognitive impairment (Forlenza et al., 2016) did not find any evidence of tauopathy. However, in another study, the total to phosphorylated tau ratio was reduced among patients with BIP carrying the risk allele of a common variant related to the previously discovered BIP risk gene *CACNA1C* (Jakobsson et al., 2016). A similar reduction was not found among healthy controls carrying the same risk allele. These findings suggest an alteration in the regulation of tau phosphorylation in carriers of the risk allele that is restricted to patients with BIP. Further studies should explore whether interactions with other genes involved in regulation of tau phosphorylation, like the *MARK2* gene, could explain the specificity of the finding to patients with BIP. Lithium has several molecular targets including inhibition of glycogen synthase kinase 3β (Freland and Beaulieu, 2012). Evidence is conflicting on whether glycogen synthase kinase 3β in turn inhibits or activates MARK2 (Kosuga et al., 2005; Timm et al., 2008). Consequently, it is unknown whether treatment with lithium could result in reduced or increased phosphorylation of tau among carriers of the common variant related to the *MARK2* gene.

The intronic variant within *VAC14* (rs11649476) was related to AD and BIP with opposite directions of effects. The same variant was shared between BIP and intelligence with concordant direction of effects in a recent study using conjunctional FDR (Smeland et al., 2019). *VAC14* encodes a part of the PIKfyve protein kinase complex, which phosphorylates phosphatidylinositol 3-phosphate [PI(3)P] to phosphatidylinositol 3,5-bisphosphate [PI(3,5)P_2_] (McCartney et al., 2014). PI(3,5)P_2_ is involved in endosomal homeostasis (Di Paolo and De Camilli, 2006). A null mutation of *VAC14* in a mouse model resulted in perinatal death and massive neurodegeneration with vacuolated neurons (Zhang et al., 2007). Amyloid precursor protein (APP) is a transmembrane protein involved in the pathophysiology of AD (O’Brien and Wong, 2011). Balklava et al. (2015) found that APP interacts with the PIKfyve complex to maintain endosomal homeostasis in *C. elegans*. They postulated that aberrant processing of APP contributes to the pathophysiology of AD through a cascade of reduced activation of PIKfyve, reduced levels of PI(3,5)P_2_, endosomal dysfunction, and reduced clearance of beta amyloid. Another example of the relationship between the processing of phosphoinositides and APP comes from a study of Miranda et al. (2018). They found that inhibition of Vps34, a kinase phosphorylating phosphatidylinositol (PI) to PI(3)P, causes endolysosomal dysfunction with secretion of exosomes containing APP C-terminal fragments. Knowles et al. (2017) recently reported that serum levels of PI, the precursor of phosphoinositides like PI(3)P and PI(3,5)P_2_, is negatively associated with a proxy of genetic susceptibility to BIP.

Some of the genes implicated by the novel loci identified by conditional FDR analyses (Supplementary Tables 1, 2 and Supplementary Figures 1, 2) also relate to known pathophysiological and clinical features of AD and BIP. The *PRKCA* gene encodes the protein kinase C alpha (PKCa). PKCa is described in amyloid plaque of patients with AD (Clark et al., 1991) where it could contribute to reduced synaptic activity (Alfonso et al., 2016). The *PRKCA* gene is higher expressed in bipolar mania compared to unipolar depression (Wang et al., 1999), and is lower expressed in fibroblasts of patients with BIP treated with lithium compared to those treated with other medications (Kittel-Schneider et al., 2016). Common genetic variants implicating the *PRKCA* gene are in healthy individuals associated to impairment of episodic memory (MacLeod and Donaldson, 2014). Variants within the *KIAA1109* gene are in family studies associated with multi-system syndromes characterized by impaired neurodevelopment (Alazami et al., 2015; Gueneau et al., 2018), while the *MTSS1L* gene is associated with neurodegeneration in a consanguineous family study (Alazami et al., 2015). The *STARD9* gene is necessary for spindle assembly during cell division in human development, and a mutation in the gene might cause a syndrome with intellectual disability (Okamoto et al., 2017). The locus implicating the *AK091365* gene was previously associated with general cognitive function when conditioned on association with schizophrenia (Smeland et al., 2017), which in turn has a high genetic correlation with BIP (Bulik-Sullivan et al., 2015). The *SSBP2* gene encodes the single strand DNA binding protein 2, which protects telomeres in a mouse model (Gu p. et al., 2013). In a Mendelian randomization study, Zhan et al. (2015) found that telomere length is causally related to AD. Telomere length is probably not reduced in most patients with BIP (Colpo et al., 2015; Darrow et al., 2016), however; one study found that patients with BIP treated with lithium had longer telomeres compared to patients not receiving lithium (Powell et al., 2017). The *RCOR2* gene product is related to cortical development (Wang et al., 2016b) and inflammation (Alvarez-López et al., 2014) in mice. The *GRIN2A* gene encodes the GluN2A subunit of the *N-*methyl-D-aspartate (NMDA) receptor. The NMDA receptor is central for synaptic plasticity and learning (Li and Tsien, 2009). Memantine, an NMDA receptor antagonist, probably reduces cognitive decline (Reisberg et al., 2003; Howard et al., 2012) and neuropsychiatric symptoms (Maidment et al., 2008) in AD. Ketamine, another NMDA receptor antagonist, can give short term remission of depression in BIP when used as an add-on to mood stabilizers (Diazgranados et al., 2010; Zarate et al., 2012). Mutations in *GRIN2A* are previously associated with a range of neuropsychiatric phenotypes including mental retardation, epilepsy, schizophrenia, and BIP (Itokawa et al., 2003; Yuan et al., 2015).

Some of the genes implicated both at genome-wide significance in previously GWASs and by conditional FDR in the present study also have pathophysiological and clinical plausibility. The expression of *TRANK1* is decreased in induced pluripotent stem cells derived neurons carrying the common variant found in our study (rs9834970). Decreased expression of *TRANK1* alters the expression of other genes related to neuronal development and differentiation (Jiang et al., 2018). Chronic treatment with sodium valproate, a mood stabilizer used in BIP (Macritchie et al., 2001), normalizes the expression of *TRANK1* (Jiang et al., 2018). The *CNTNAP5* gene encodes a transmembrane protein of the neurexin family, which is related to cellular adhesion and intercellular communication (Traut et al., 2006). Common variants implicating *CNTNAP5* have previously been associated with the posterior cortical atrophy variant of AD (Schott et al., 2016), BIP (Djurovic et al., 2010), and response to antipsychotic treatment in schizophrenia (Yu et al., 2018), while rare variants within *CNTNAP5* have previously been associated with autism spectrum disorders (Pagnamenta et al., 2010). The *NCAN* gene is involved in neuronal adhesion and migration (Raum et al., 2015). Common variants implicating *NCAN* are associated with cognitive performance (Raum et al., 2015) and limbic gray matter volumes (Dannlowski et al., 2015) in healthy individuals, while a rare variant is associated with dyslexia (Einarsdottir et al., 2017).

Further experimental studies should examine the implications of our findings. It is unknown if the loci implicated by condFDR and conjFDR relate to altered levels of gene expression, pathophysiological processes (e.g., impaired neuronal migration, tauopathy, and disturbed endosomal homeostasis), clinical features (e.g., cognitive and affective symptoms), and treatment response to lithium among patients with AD and BIP. Further, it is unknown if the loci interact with environmental risk factors and other genes implicated in AD and BIP.

Our results should be interpreted in light of the following limitations. We can neither exclude that some of the patients with AD have had BIP, nor that some of the patients with BIP will develop AD, which could have confounded our results. However; this could not explain the finding in the conjunctional FDR analyses of one locus implicated in AD and BIP with opposite directions of effect. Due to linkage disequilibrium among SNPs, our findings do not necessarily reflect causal variants, or that the same causal variants are involved in both traits. Although we found indications of modest polygenic overlap using conditional QQ-plots (Figure 1), we only detected two genetic loci jointly associated with both AD and BIP (Figure 2). However, the observed enrichment suggests that more shared SNPs will be identified when GWAS sample sizes increase (Schork et al., 2016). Further, we have only assessed the shared common genetic variants between AD and BIP. Other genetic variations, like rare structural variants, are also shown to increase the risk of AD and BIP (Lord et al., 2014; Cruceanu et al., 2017). Lastly, most participants in the data used in our study are of European ancestry. The generalizability of our findings to samples dominated by participants of other ancestries is unknown.

## Conclusion

We find polygenic overlap between AD and BIP and identify novel loci associated with each trait and jointly with both traits, providing new insights into their genetic architecture. The genes *MARK2* and *VAC14* jointly implicated in AD and BIP are previously described to be involved in neuronal migration, tau phosphorylation, and endosomal homeostasis. Further experimental studies should examine if our findings translate to altered levels of transcription, pathophysiological processes, clinical features, and treatment response to lithium among patients with AD and BIP.

## Author Contributions

OD, PF, SH, and OA designed the protocol of the study. OA and OS obtained funding. AS, OS, OF, SH, and YW conducted the analyses. OS, AS, AW, OF, YW, SH, and OA interpreted the results. OD, OS, and AS drafted the manuscript. All authors contributed with the further writing of the manuscript and approved the final manuscript.

## Conflict of Interest Statement

OA has received a speaker’s honorarium from Lundbeck and has a patent application (US 20150356243) pending. AD also applied for this patent application and assigned it to UC San Diego. AD has additional disclosures outside the present work: founder, equity holder, and advisory board member for CorTechs Labs, advisory board member of Human Longevity, recipient of non-financial research support from General Electric Healthcare. The remaining authors declare that the research was conducted in the absence of any commercial or financial relationships that could be construed as a potential conflict of interest.

## Footnotes

1 https://github.com/Nealelab/ricopili/wiki

2 http://web.pasteur-lille.fr/en/recherche/u744/igap/igap_download.php

3 https://www.med.unc.edu/pgc/results-and-downloads

4 http://hbatlas.org

## Abbreviations

AD: Alzheimer’s disease
BIP: bipolar disorder
FDR: false discovery rate
GWAS: genome wide association study
IGAP: International Genomics of Alzheimer’s Project
LD: linkage disequilibrium
LDSR: Linkage disequilibrium score regression
PGC2-BIP: Psychiatric Genetic Consortium 2 Bipolar Disorder Working Group
SNP: single nucleotide polymorphism
QQ: quantile-quantile
