# Supplementary material for: Genetic Overlap Between Alzheimer’s Disease and Bipolar Disorder Implicates the MARK2 and VAC14 Genes
Source: Front Neurosci. 2019 Mar 13;13:220. doi: 10.3389/fnins.2019.00220 (PMC6425305; doi:10.3389/fnins.2019.00220)
Supplement: Supplementary file 1 [file Data_Sheet_1.docx]

*Supplementary Materials*

Genetic Overlap Between Alzheimer’s Disease and Bipolar Disorder Implicates the MARK2 and VAC14 Genes

**Ole Kristian Drange^*^, Olav Bjerkehagen Smeland, Alexey A. Shadrin, Per Ivar Finseth, Aree Witoelar, Oleksandr Frei, Psychiatric Genomics Consortium Bipolar Disorder Working Group, Yunpeng Wang, Sahar Hassani, Srdjan Djurovic, Anders M. Dale, Ole A. Andreassen**

*** Correspondence:** Ole Kristian Drange: [ole.kristian.drange@gmail.com](mailto:ole.kristian.drange@gmail.com)

[1 Supplementary methods 2](#_Toc505022343)

[1.1 Conditional QQ-plots 2](#_Toc505022344)

[1.2 Conditional false discovery rate 3](#_Toc505022345)

[1.3 Conjunctional false discovery rate 4](#_Toc505022346)

[1.4 Cross-trait linkage disequilibrium score regression 5](#_Toc505022347)

[1.5 The Psychiatric Genetic Consortium 2 Bipolar Disorder Working Group authors 6](#_Toc505022348)

[2 Supplementary tables 13](#_Toc505022349)

[3 Supplementary figures 15](#_Toc505022350)

[4 Supplementary references 19](#_Toc505022351)

# Supplementary methods

## Conditional QQ-plots

A conditional Q-Q plot compares the quantiles of two probability distributions. We calculated the empirical cumulative distribution function (ecdf) of nominal p-values of all SNPs for the first trait. For the second trait, we stratified SNPs according to defined p-value thresholds; p <1 (all SNPs), <10^-1^, <10^-2^ and <10^-3^. We then plotted the nominal p-values, denoted *p*, on the y-axis, and 1-ecdf, denoted *q*, on the x-axis. To emphasize the tail probabilities of both distributions, we transformed the y and x axes to –log_10_(p) and –log_10_(q). This procedure was conducted with AD as the first and BIP as the second trait and *vice versa*.

Pleiotropic enrichment is present if the degree of leftward shift from the expected null line for the first trait is dependent on the degree of association with the second trait. A leftward shift from the expected null line in Q-Q plots translates to a reduction in the false discovery rate (FDR), as explained in the next section.

## Conditional false discovery rate

We translated the enrichment observed in QQ-plots to conditional false discovery rate (FDR) as outlined by Andreassen et al. (2013). A given p-value cut-off relates to FDR according to the equation

1. $\mathrm{FDR}\left( p \right)=\frac{\pi_{0}F_{0}\left( p \right)}{F\left( p \right)},$

where π_0_ denotes the proportion of null SNPs, F_0_ denotes the null cumulative distribution function (cdf), and F(p) denotes the cdf of all SNPs. π_0_ is in GWAS of most complex traits close to one. F_0_ is a standard uniform distribution given that all intervals of the cdf are equally probable under the null hypothesis. F(p) is estimated by the empirical cdf, denoted q, by the equation

1. $q=\frac{N_{p}}{N},$

where N_p_ denotes the number of SNPs with p-values < p, and N denotes the total number of SNPs. Incorporating these assumptions into the equation (1) gives

1. $\mathrm{FDR}\left( p \right)\approx\frac{p}{q}.$

By conducting a logarithmic transformation and dividing by minus one on each side of equation (3) we get

1. $-\log_{10}\left( \mathrm{FDR}\left( p \right) \right)\approx\log_{10}\left( q \right)-\log_{10}(p),$

which demonstrates that the horizontal shift in QQ-plots (i.e. an increase in q relative to p) relates negatively to FDR. The conditional FDR is defined as “the posterior probability that a given SNP is null for the first trait given that the p-values for both traits are as small or smaller than the observed p-values” (Andreassen et al., 2015), as given by

1. $\mathrm{FDR}\left( p_{1} | p_{2} \right)=\frac{\pi_{0}p_{2}\left( p_{1} \right)}{F\left( p_{1}|p_{2} \right)}$,

where p_1_ denotes the p-value of one trait, p_2_ denotes the p-value the other trait, F(p_1_|p_2_) denotes the conditional cdf, and π_0_(p_2_) denotes the conditional proportion of null SNPs for one trait given that the p-values for the other trait are < p_2_. The conditional FDR is conservatively estimated if π_0_(p_2_) is set to 0. F(p_1_|p_2_) can be replaced by the empirical conditional cdf. Thus, we can calculate the conditional FDR value for one trait, trait_1_, conditioned on p-values for another trait, trait_2_, for each SNP. The values are denoted condFDR_(trait1|trait2)_.

## Conjunctional false discovery rate

The conjunctional FDR is defined as the “the posterior probability that a given SNP is null for the first trait given that the p-values for both traits are as small or smaller than the observed p-values” (Andreassen et al., 2015), as given by

1. $\mathrm{FDR}\left( p_{1},p_{2} \right)=\pi_{0}(p_{1},p_{2})F_{0}(p_{1},p_{2})/F(p_{1},p_{2})$,

where π_0_(p_1_,p_2_) denotes the proportion of SNPs null for both traits at the same time, F_0_(p_1_,p_2_) denotes the joint null cfd, and F(p_1_,p_2_) denotes the joint cfd. Equation (6) can be conservatively estimated by conditional empirical cdfs formulated by

1. $\mathrm{FDR}_{trait1\&trait2}=max\left\{ \mathrm{FDR}_{trait1|trait2},\mathrm{FDR}_{trait2|trait1} \right\},$

as explained by Andreassen et al. (2013). Thus, we can calculate the conjuntional FDR value for both traits for each SNP. These values are denoted conjFDR_(trait1&trait2)_.

## Cross-trait linkage disequilibrium score regression

In the case of an inflated signal in test statistics from a GWAS, single-trait linkage disequilibrium score regression (LDSR) distinguishes the effect of polygenicity from the effect of confounding (e.g. from population stratification) (Bulik-Sullivan et al., 2015b). Cross-trait LDSR is a simple extension from single-trait LDSR that estimates the overall degree of genetic overlap between two traits (Bulik-Sullivan et al., 2015a).

## The Psychiatric Genetic Consortium 2 Bipolar Disorder Working Group authors

Eli A Stahl^1,2,3†&,^ Gerome Breen^4,5†^, Andreas J Forstner^6,7,8,9,10†,^ Andrew McQuillin^11†,^ Stephan Ripke^12,13,14†^, Vassily Trubetskoy^13^, Manuel Mattheisen^15,16,17,18,19^, Yunpeng Wang^20,21^, Jonathan R I Coleman^4,5^, Héléna A Gaspar^4,5^, Christiaan A de Leeuw^22^, Stacy Steinberg^23^, Jennifer M Whitehead Pavlides^24^, Maciej Trzaskowski^25^, Tune H Pers^3,26^, Peter A Holmans^27^, Liam Abbott^12^, Esben Agerbo^19,28,29^, Huda Akil^30^, Diego Albani^31^, Ney Alliey-Rodriguez^32^, Thomas D Als^15,16,19^, Adebayo Anjorin^33^, Verneri Antilla^14^, Swapnil Awasthi^13^, Judith A Badner^34^, Marie Bækvad-Hansen^19,35^, Jack D Barchas^36^, Nicholas Bass^11^, Michael Bauer^37^, Richard Belliveau^12^, Sarah E Bergen^38^, Carsten Bøcker Pedersen^19,28,29^, Erlend Bøen^39^, Marco Boks^40^, James Boocock^41^, Monika Budde^42^, William Bunney^43^, Margit Burmeister^44^, Jonas Bybjerg-Grauholm^19,35^, William Byerley^45^, Miquel Casas^46,47,48,49^, Felecia Cerrato^12^, Pablo Cervantes^50^, Kimberly Chambert^12^, Alexander W Charney^2^, Danfeng Chen^12^, Claire Churchhouse^12,14^, Toni-Kim Clarke^51^, William Coryell^52^, David W Craig^53^, Cristiana Cruceanu^50,54^, David Curtis^55,56^, Piotr M Czerski^57^, Anders M Dale^58,59,60,61^, Simone de Jong^4,5^, Franziska Degenhardt^8,9^, Jurgen Del-Favero^62^, J Raymond DePaulo^63^, Srdjan Djurovic^64,65^, Amanda L Dobbyn^1,2^, Ashley Dumont^12^, Torbjørn Elvsåshagen^66,67^, Valentina Escott-Price^27^, Chun Chieh Fan^61^, Sascha B Fischer^6,10^, Matthew Flickinger^68^, Tatiana M Foroud^69^, Liz Forty^27^, Josef Frank^70^, Christine Fraser^27^, Nelson B Freimer^71^, Louise Frisén^72,73,74^, Katrin Gade^42,75^, Diane Gage^12^, Julie Garnham^76^, Claudia Giambartolomei^41^, Marianne Giørtz Pedersen^19,28,29^, Jaqueline Goldstein^12^, Scott D Gordon^77^, Katherine Gordon-Smith^78^, Elaine K Green^79^, Melissa J Green^80^, Tiffany A Greenwood^60^, Jakob Grove^15,16,19,81^, Weihua Guan^82^, José Guzman Parra^83^, Marian L Hamshere^27^, Martin Hautzinger^84^, Urs Heilbronner^42^, Stefan Herms^6,8,9,10^, Maria Hipolito^85^, Per Hoffmann^6,8,9,10^, Dominic Holland^58,86^, Laura Huckins^1,2^, Stéphane Jamain^87,88^, Jessica S Johnson^1,2^, Anders Juréus^38^, Radhika Kandaswamy^4^, Robert Karlsson^38^, James L Kennedy^89,90,91,92^, Sarah Kittel-Schneider^93^, Sarah V Knott^78^, James A Knowles^94,95^, Manolis Kogevinas^96^, Anna C Koller^8,9^, Ralph Kupka^97,98,99^, Catharina Lavebratt^72^, Jacob Lawrence^100^, William B Lawson^85^, Markus Leber^101^, Phil H Lee^12,14,102^, Shawn E Levy^103^, Jun Z Li^104^, Chunyu Liu^105^, Susanne Lucae^106^, Anna Maaser^8,9^, Donald J MacIntyre^107,108^, Pamela B Mahon^63,109^, Wolfgang Maier^110^, Lina Martinsson^73^, Steve McCarroll^12,111^, Peter McGuffin^4^, Melvin G McInnis^112^, James D McKay^113^, Helena Medeiros^95^, Sarah E Medland^77^, Fan Meng^30,112^, Lili Milani^114^, Grant W Montgomery^25^, Derek W Morris^115,116^, Thomas W Mühleisen^6,117^, Niamh Mullins^4^, Hoang Nguyen^1,2^, Caroline M Nievergelt^60,118^, Annelie Nordin Adolfsson^119^, Evaristus A Nwulia^85^, Claire O'Donovan^76^, Loes M Olde Loohuis^71^, Anil P S Ori^71^, Lilijana Oruc^120^, Urban Ösby^121^, Roy H Perlis^122,123^, Amy Perry^78^, Andrea Pfennig^37^, James B Potash^63^, Shaun M Purcell^2,109^, Eline J Regeer^124^, Andreas Reif^93^, Céline S Reinbold^6,10^, John P Rice^125^, Fabio Rivas^83^, Margarita Rivera^4,126^, Panos Roussos^1,2,127^, Douglas M Ruderfer^128^, Euijung Ryu^129^, Cristina Sánchez-Mora^46,47,49^, Alan F Schatzberg^130^, William A Scheftner^131^, Nicholas J Schork^132^, Cynthia Shannon Weickert^80,133^, Tatyana Shehktman^60^, Paul D Shilling^60^, Engilbert Sigurdsson^134^, Claire Slaney^76^, Olav B Smeland^58,135,136^, Janet L Sobell^137^, Christine Søholm Hansen^19,35^, Anne T Spijker^138^, David St Clair^139^, Michael Steffens^140^, John S Strauss^91,141^, Fabian Streit^70^, Jana Strohmaier^70^, Szabolcs Szelinger^142^, Robert C Thompson^112^, Thorgeir E Thorgeirsson^23^, Jens Treutlein^70^, Helmut Vedder^143^, Weiqing Wang^1,2^, Stanley J Watson^112^, Thomas W Weickert^80,133^, Stephanie H Witt^70^, Simon Xi^144^, Wei Xu^145,146^, Allan H Young^147^, Peter Zandi^148^, Peng Zhang^149^, Sebastian Zollner^112^, Rolf Adolfsson^119^, Ingrid Agartz^17,39,150^, Martin Alda^76,151^, Lena Backlund^73^, Bernhard T Baune^152^, Frank Bellivier^153,154,155,156^, Wade H Berrettini^157^, Joanna M Biernacka^129^, Douglas H R Blackwood^51^, Michael Boehnke^68^, Anders D Børglum^15,16,19^, Aiden Corvin^116^, Nicholas Craddock^27^, Mark J Daly^12,14^, Udo Dannlowski^158^, Tõnu Esko^3,111,114,159^, Bruno Etain^153,155,156,160^, Mark Frye^161^, Janice M Fullerton^133,162^, Elliot S Gershon^32,163^, Michael Gill^116^, Fernando Goes^63^, Maria Grigoroiu-Serbanescu^164^, Joanna Hauser^57^, David M Hougaard^19,35^, Christina M Hultman^38^, Ian Jones^27^, Lisa A Jones^78^, René S Kahn^2,40^, George Kirov^27^, Mikael Landén^38,165^, Marion Leboyer^88,153,166^, Cathryn M Lewis^4,5,167^, Qingqin S Li^168^, Jolanta Lissowska^169^, Nicholas G Martin^77,170^, Fermin Mayoral^83^, Susan L McElroy^171^, Andrew M McIntosh^51,172^, Francis J McMahon^173^, Ingrid Melle^174,175^, Andres Metspalu^114,176^, Philip B Mitchell^80^, Gunnar Morken^177,178^, Ole Mors^19,179^, Preben Bo Mortensen^15,19,28,29^, Bertram Müller-Myhsok^54,180,181^, Richard M Myers^103^, Benjamin M Neale^3,12,14^, Vishwajit Nimgaonkar^182^, Merete Nordentoft^19,183^, Markus M Nöthen^8,9^, Michael C O'Donovan^27^, Ketil J Oedegaard^184,185^, Michael J Owen^27^, Sara A Paciga^186^, Carlos Pato^95,187^, Michele T Pato^95^, Danielle Posthuma^22,188^, Josep Antoni Ramos-Quiroga^46,47,48,49^, Marta Ribasés^46,47,49^, Marcella Rietschel^70^, Guy A Rouleau^189,190^, Martin Schalling^72^, Peter R Schofield^133,162^, Thomas G Schulze^42,63,70,75,173^, Alessandro Serretti^191^, Jordan W Smoller^12,192,193^, Hreinn Stefansson^23^, Kari Stefansson^23,194^, Eystein Stordal^195,196^, Patrick F Sullivan^38,197,198^, Gustavo Turecki^199^, Arne E Vaaler^200^, Eduard Vieta^201^, John B Vincent^141^, Thomas Werge^19,202,203^, John I Nurnberger^204^, Naomi R Wray^24,25^, Arianna Di Florio^27,198^, Howard J Edenberg^205^, Sven Cichon^6,8,10,117^, Roel A Ophoff^40,41,71^, Laura J Scott^68^, Ole A Andreassen^135,136^, John Kelsoe^60*^, Pamela Sklar^1,2*^^

† Equal contribution * Co-last authors ^ deceased

Affiliations:

1 Department of Genetics and Genomic Sciences, Icahn School of Medicine at Mount Sinai, New York, NY, US

2 Department of Psychiatry, Icahn School of Medicine at Mount Sinai, New York, NY, US

3 Medical and Population Genetics, Broad Institute, Cambridge, MA, US

4 MRC Social, Genetic and Developmental Psychiatry Centre, King's College London, London, GB

5 NIHR BRC for Mental Health, King's College London, London, GB

6 Department of Biomedicine, University of Basel, Basel, CH

7 Department of Psychiatry (UPK), University of Basel, Basel, CH

8 Institute of Human Genetics, University of Bonn, Bonn, DE

9 Life&Brain Center, Department of Genomics, University of Bonn, Bonn, DE

10 Institute of Medical Genetics and Pathology, University Hospital Basel, Basel, CH

11 Division of Psychiatry, University College London, London, GB

12 Stanley Center for Psychiatric Research, Broad Institute, Cambridge, MA, US

13 Department of Psychiatry and Psychotherapy, Charité - Universitätsmedizin, Berlin, DE

14 Analytic and Translational Genetics Unit, Massachusetts General Hospital, Boston, MA, US

15 iSEQ, Center for Integrative Sequencing, Aarhus University, Aarhus, DK

16 Department of Biomedicine - Human Genetics, Aarhus University, Aarhus, DK

17 Department of Clinical Neuroscience, Centre for Psychiatry Research, Karolinska Institutet, Stockholm, SE

18 Department of Psychiatry, Psychosomatics and Psychotherapy, Center of Mental Health, University Hospital Würzburg, Würzburg, DE

19 iPSYCH, The Lundbeck Foundation Initiative for Integrative Psychiatric Research, DK

20 Institute of Biological Psychiatry, Mental Health Centre Sct. Hans, Copenhagen, DK

21 Institute of Clinical Medicine, University of Oslo, Oslo, NO

22 Department of Complex Trait Genetics, Center for Neurogenomics and Cognitive Research, Amsterdam Neuroscience, Vrije Universiteit Amsterdam, Amsterdam, NL

23 deCODE Genetics / Amgen, Reykjavik, IS

24 Queensland Brain Institute, The University of Queensland, Brisbane, QLD, AU

25 Institute for Molecular Bioscience, The University of Queensland, Brisbane, QLD, AU

26 Division of Endocrinology and Center for Basic and Translational Obesity Research, Boston Children’s Hospital, Boston, MA, US

27 Medical Research Council Centre for Neuropsychiatric Genetics and Genomics, Division of Psychological Medicine and Clinical Neurosciences, Cardiff University, Cardiff, GB

28 National Centre for Register-Based Research, Aarhus University, Aarhus, DK

29 Centre for Integrated Register-based Research, Aarhus University, Aarhus, DK

30 Molecular & Behavioral Neuroscience Institute, University of Michigan, Ann Arbor, MI, US

31 NEUROSCIENCE, Istituto Di Ricerche Farmacologiche Mario Negri, Milano, IT

32 Department of Psychiatry and Behavioral Neuroscience, University of Chicago, Chicago, IL, US

33 Psychiatry, Berkshire Healthcare NHS Foundation Trust, Bracknell, GB

34 Psychiatry, Rush University Medical Center, Chicago, IL, US

35 Center for Neonatal Screening, Department for Congenital Disorders, Statens Serum Institut, Copenhagen, DK

36 Department of Psychiatry, Weill Cornell Medical College, New York, NY, US

37 Department of Psychiatry and Psychotherapy, University Hospital Carl Gustav Carus, Technische Universität Dresden, Dresden, DE

38 Department of Medical Epidemiology and Biostatistics, Karolinska Institutet, Stockholm, SE

39 Department of Psychiatric Research, Diakonhjemmet Hospital, Oslo, NO

40 Psychiatry, UMC Utrecht Hersencentrum Rudolf Magnus, Utrecht, NL

41 Human Genetics, University of California Los Angeles, Los Angeles, CA, US

42 Institute of Psychiatric Phenomics and Genomics (IPPG), University Hospital, LMU Munich, Munich, DE

43 Department of Psychiatry and Human Behavior, University of California, Irvine, Irvine, CA, US

44 Molecular & Behavioral Neuroscience Institute and Department of Computational Medicine & Bioinformatics, University of Michigan, Ann Arbor, MI, US

45 Psychiatry, University of California San Francisco, San Francisco, CA, US

46 Instituto de Salud Carlos III, Biomedical Network Research Centre on Mental Health (CIBERSAM), Madrid, ES

47 Department of Psychiatry, Hospital Universitari Vall d ́Hebron, Barcelona, ES

48 Department of Psychiatry and Forensic Medicine, Universitat Autònoma de Barcelona, Barcelona, ES

49 Psychiatric Genetics Unit, Group of Psychiatry Mental Health and Addictions, Vall d ́Hebron Research Institut (VHIR), Universitat Autònoma de Barcelona, Barcelona, ES

50 Department of Psychiatry, Mood Disorders Program, McGill University Health Center, Montreal, QC, CA

51 Division of Psychiatry, University of Edinburgh, Edinburgh, GB

52 University of Iowa Hospitals and Clinics, Iowa City, IA, US

53 Translational Genomics, USC, Phoenix, AZ, US

54 Department of Translational Research in Psychiatry, Max Planck Institute of Psychiatry, Munich, DE

55 Centre for Psychiatry, Queen Mary University of London, London, GB

56 UCL Genetics Institute, University College London, London, GB

57 Department of Psychiatry, Laboratory of Psychiatric Genetics, Poznan University of Medical Sciences, Poznan, PL

58 Department of Neurosciences, University of California San Diego, La Jolla, CA, US

59 Department of Radiology, University of California San Diego, La Jolla, CA, US

60 Department of Psychiatry, University of California San Diego, La Jolla, CA, US

61 Department of Cognitive Science, University of California San Diego, La Jolla, CA, US

62 Applied Molecular Genomics Unit, VIB Department of Molecular Genetics, University of Antwerp, Antwerp, Belgium

63 Department of Psychiatry and Behavioral Sciences, Johns Hopkins University School of Medicine, Baltimore, MD, US

64 Department of Medical Genetics, Oslo University Hospital Ullevål, Oslo, NO

65 NORMENT, KG Jebsen Centre for Psychosis Research, Department of Clinical Science, University of Bergen, Bergen, NO

66 Department of Neurology, Oslo University Hospital, Oslo, NO

67 NORMENT, KG Jebsen Centre for Psychosis Research, Oslo University Hospital, Oslo, NO

68 Center for Statistical Genetics and Department of Biostatistics, University of Michigan, Ann Arbor, MI, US

69 Department of Medical & Molecular Genetics, Indiana University, Indianapolis, IN, US

70 Department of Genetic Epidemiology in Psychiatry, Central Institute of Mental Health, Medical Faculty Mannheim, Heidelberg University, Mannheim, DE

71 Center for Neurobehavioral Genetics, University of California Los Angeles, Los Angeles, CA, US

72 Department of Molecular Medicine and Surgery, Karolinska Institutet and Center for Molecular Medicine, Karolinska University Hospital, Stockholm, SE

73 Department of Clinical Neuroscience, Karolinska Institutet and Center for Molecular Medicine, Karolinska University Hospital, Stockholm, SE

74 Child and Adolescent Psychiatry Research Center, Stockholm, SE

75 Department of Psychiatry and Psychotherapy, University Medical Center Göttingen, Göttingen, DE

76 Department of Psychiatry, Dalhousie University, Halifax, NS, CA

77 Genetics and Computational Biology, QIMR Berghofer Medical Research Institute, Brisbane, QLD, AU

78 Department of Psychological Medicine, University of Worcester, Worcester, GB

79 School of Biomedical and Healthcare Sciences, Plymouth University Peninsula Schools of Medicine and Dentistry, Plymouth, GB

80 School of Psychiatry, University of New South Wales, Sydney, NSW, AU

81 Bioinformatics Research Centre, Aarhus University, Aarhus, DK

82 Biostatistics, University of Minnesota System, Minneapolis, MN, US

83 Mental Health Department, University Regional Hospital, Biomedicine Institute (IBIMA), Málaga, ES

84 Department of Psychology, Eberhard Karls Universität Tübingen, Tubingen, DE

85 Department of Psychiatry and Behavioral Sciences, Howard University Hospital, Washington, DC, US

86 Center for Multimodal Imaging and Genetics, University of California San Diego, La Jolla, CA, US

87 Psychiatrie Translationnelle, Inserm U955, Créteil, FR

88 Faculté de Médecine, Université Paris Est, Créteil, FR

89 Campbell Family Mental Health Research Institute, Centre for Addiction and Mental Health, Toronto, ON, CA

90 Neurogenetics Section, Centre for Addiction and Mental Health, Toronto, ON, CA

91 Department of Psychiatry, University of Toronto, Toronto, ON, CA

92 Institute of Medical Sciences, University of Toronto, Toronto, ON, CA

93 Department of Psychiatry, Psychosomatic Medicine and Psychotherapy, University Hospital Frankfurt, Frankfurt am Main, DE

94 Cell Biology, SUNY Downstate Medical Center College of Medicine, Brooklyn, NY, US

95 Institute for Genomic Health, SUNY Downstate Medical Center College of Medicine, Brooklyn, NY, US

96 Center for Research in Environmental Epidemiology (CREAL), Barcelona, ES

97 Psychiatry, Altrecht, Utrecht, NL

98 Psychiatry, GGZ inGeest, Amsterdam, NL

99 Psychiatry, VU medisch centrum, Amsterdam, NL

100 Psychiatry, North East London NHS Foundation Trust, Ilford, GB

101 Clinic for Psychiatry and Psychotherapy, University Hospital Cologne, Cologne, DE

102 Psychiatric and Neurodevelopmental Genetics Unit, Massachusetts General Hospital, Boston, MA, US

103 HudsonAlpha Institute for Biotechnology, Huntsville, AL, US

104 Department of Human Genetics, University of Michigan, Ann Arbor, MI, US

105 Psychiatry, University of Illinois at Chicago College of Medicine, Chicago, IL, US

106 Max Planck Institute of Psychiatry, Munich, DE

107 Mental Health, NHS 24, Glasgow, GB

108 Division of Psychiatry, Centre for Clinical Brain Sciences, University of Edinburgh, Edinburgh, GB

109 Psychiatry, Brigham and Women's Hospital, Boston, MA, US

110 Department of Psychiatry and Psychotherapy, University of Bonn, Bonn, DE

111 Department of Genetics, Harvard Medical School, Boston, MA, US

112 Department of Psychiatry, University of Michigan, Ann Arbor, MI, US

113 Genetic Cancer Susceptibility Group, International Agency for Research on Cancer, Lyon, FR

114 Estonian Genome Center, University of Tartu, Tartu, EE

115 Discipline of Biochemistry, Neuroimaging and Cognitive Genomics (NICOG) Centre, National University of Ireland, Galway, Galway, IE

116 Neuropsychiatric Genetics Research Group, Dept of Psychiatry and Trinity Translational Medicine Institute, Trinity College Dublin, Dublin, IE

117 Institute of Neuroscience and Medicine (INM-1), Research Centre Jülich, Jülich, DE

118 Research/Psychiatry, Veterans Affairs San Diego Healthcare System, San Diego, CA, US

119 Department of Clinical Sciences, Psychiatry, Umeå University Medical Faculty, Umeå, SE

120 Department of Clinical Psychiatry, Psychiatry Clinic, Clinical Center University of Sarajevo, Sarajevo, BA

121 Department of Neurobiology, Care sciences, and Society, Karolinska Institutet and Center for Molecular Medicine, Karolinska University Hospital, Stockholm, SE

122 Psychiatry, Harvard Medical School, Boston, MA, US

123 Division of Clinical Research, Massachusetts General Hospital, Boston, MA, US

124 Outpatient Clinic for Bipolar Disorder, Altrecht, Utrecht, NL

125 Department of Psychiatry, Washington University in Saint Louis, Saint Louis, MO, US

126 Department of Biochemistry and Molecular Biology II, Institute of Neurosciences, Center for Biomedical Research, University of Granada, Granada, ES

127 Department of Neuroscience, Icahn School of Medicine at Mount Sinai, New York, NY, US

128 Medicine, Psychiatry, Biomedical Informatics, Vanderbilt University Medical Center, Nashville, TN, US

129 Department of Health Sciences Research, Mayo Clinic, Rochester, MN, US

130 Psychiatry and Behavioral Sciences, Stanford University School of Medicine, Stanford, CA, US

131 Rush University Medical Center, Chicago, IL, US

132 Scripps Translational Science Institute, La Jolla, CA, US

133 Neuroscience Research Australia, Sydney, NSW, AU

134 Faculty of Medicine, Department of Psychiatry, School of Health Sciences, University of Iceland, Reykjavik, IS

135 Div Mental Health and Addiction, Oslo University Hospital, Oslo, NO

136 NORMENT, University of Oslo, Oslo, NO

137 Psychiatry and the Behavioral Sciences, University of Southern California, Los Angeles, CA, US

138 Mood Disorders, PsyQ, Rotterdam, NL

139 Institute for Medical Sciences, University of Aberdeen, Aberdeen, UK

140 Research Division, Federal Institute for Drugs and Medical Devices (BfArM), Bonn, DE

141 Centre for Addiction and Mental Health, Toronto, ON, CA

142 Neurogenomics, TGen, Los Angeles, AZ, US

143 Psychiatry, Psychiatrisches Zentrum Nordbaden, Wiesloch, DE

144 Computational Sciences Center of Emphasis, Pfizer Global Research and Development, Cambridge, MA, US

145 Department of Biostatistics, Princess Margaret Cancer Centre, Toronto, ON, CA

146 Dalla Lana School of Public Health, University of Toronto, Toronto, ON, CA

147 Psychological Medicine, Institute of Psychiatry, Psychology & Neuroscience, King's College London, London, GB

148 Department of Mental Health, Johns Hopkins University Bloomberg School of Public Health, Baltimore, MD, US

149 Institute of Genetic Medicine, Johns Hopkins University School of Medicine, Baltimore, MD, US

150 NORMENT, KG Jebsen Centre for Psychosis Research, Division of Mental Health and Addiction, Institute of Clinical Medicine and Diakonhjemmet Hospital, University of Oslo, Oslo, NO

151 National Institute of Mental Health, Klecany, CZ

152 Discipline of Psychiatry, University of Adelaide, Adelaide, SA, AU

153 Department of Psychiatry and Addiction Medicine, Assistance Publique - Hôpitaux de Paris, Paris, FR

154 Paris Bipolar and TRD Expert Centres, FondaMental Foundation, Paris, FR

155 UMR-S1144 Team 1: Biomarkers of relapse and therapeutic response in addiction and mood disorders, INSERM, Paris, FR

156 Psychiatry, Université Paris Diderot, Paris, FR

157 Psychiatry, University of Pennsylvania, Philadelphia, PA, US

158 Department of Psychiatry, University of Münster, Münster, DE

159 Division of Endocrinology, Children's Hospital Boston, Boston, MA, US

160 Centre for Affective Disorders, Institute of Psychiatry, Psychology and Neuroscience, London, GB

161 Department of Psychiatry & Psychology, Mayo Clinic, Rochester, MN, US

162 School of Medical Sciences, University of New South Wales, Sydney, NSW, AU

163 Department of Human Genetics, University of Chicago, Chicago, IL, US

164 Biometric Psychiatric Genetics Research Unit, Alexandru Obregia Clinical Psychiatric Hospital, Bucharest, RO

165 Institute of Neuroscience and Physiology, University of Gothenburg, Gothenburg, SE

166 INSERM, Paris, FR

167 Department of Medical & Molecular Genetics, King's College London, London, GB

168 Neuroscience Therapeutic Area, Janssen Research and Development, LLC, Titusville, NJ, US

169 Cancer Epidemiology and Prevention, M. Sklodowska-Curie Cancer Center and Institute of Oncology, Warsaw, PL

170 School of Psychology, The University of Queensland, Brisbane, QLD, AU

171 Research Institute, Lindner Center of HOPE, Mason, OH, US

172 Centre for Cognitive Ageing and Cognitive Epidemiology, University of Edinburgh, Edinburgh, GB

173 Human Genetics Branch, Intramural Research Program, National Institute of Mental Health, Bethesda, MD, US

174 Division of Mental Health and Addiction, Oslo University Hospital, Oslo, NO

175 Division of Mental Health and Addiction, University of Oslo, Institute of Clinical Medicine, Oslo, NO

176 Institute of Molecular and Cell Biology, University of Tartu, Tartu, EE

177 Mental Health, Faculty of Medicine and Health Sciences, Norwegian University of Science and Technology - NTNU, Trondheim, NO

178 Psychiatry, St Olavs University Hospital, Trondheim, NO

179 Psychosis Research Unit, Aarhus University Hospital, Risskov, DK

180 Munich Cluster for Systems Neurology (SyNergy), Munich, DE

181 University of Liverpool, Liverpool, GB

182 Psychiatry and Human Genetics, University of Pittsburgh, Pittsburgh, PA, US

183 Mental Health Services in the Capital Region of Denmark, Mental Health Center Copenhagen, University of Copenhagen, Copenhagen, DK

184 Division of Psychiatry, Haukeland Universitetssjukehus, Bergen, NO

185 Faculty of Medicine and Dentistry, University of Bergen, Bergen, NO

186 Human Genetics and Computational Biomedicine, Pfizer Global Research and Development, Groton, CT, US

187 College of Medicine Institute for Genomic Health, SUNY Downstate Medical Center College of

Medicine, Brooklyn, NY, US

188 Department of Clinical Genetics, Amsterdam Neuroscience, Vrije Universiteit Medical Center,

Amsterdam, NL

189 Department of Neurology and Neurosurgery, McGill University, Faculty of Medicine, Montreal, QC, CA

190 Montreal Neurological Institute and Hospital, Montreal, QC, CA

191 Department of Biomedical and NeuroMotor Sciences, University of Bologna, Bologna, IT

192 Department of Psychiatry, Massachusetts General Hospital, Boston, MA, US

193 Psychiatric and Neurodevelopmental Genetics Unit (PNGU), Massachusetts General Hospital, Boston, MA, US

194 Faculty of Medicine, University of Iceland, Reykjavik, IS

195 Department of Psychiatry, Hospital Namsos, Namsos, NO

196 Department of Neuroscience, Norges Teknisk Naturvitenskapelige Universitet Fakultet for

naturvitenskap og teknologi, Trondheim, NO

197 Department of Genetics, University of North Carolina at Chapel Hill, Chapel Hill, NC, US

198 Department of Psychiatry, University of North Carolina at Chapel Hill, Chapel Hill, NC, US

199 Department of Psychiatry, McGill University, Montreal, QC, CA

200 Dept of Psychiatry, Sankt Olavs Hospital Universitetssykehuset i Trondheim, Trondheim, NO

201 Clinical Institute of Neuroscience, Hospital Clinic, University of Barcelona, IDIBAPS, CIBERSAM, Barcelona, ES

202 Institute of Biological Psychiatry, MHC Sct. Hans, Mental Health Services Copenhagen, Roskilde, DK

203 Department of Clinical Medicine, University of Copenhagen, Copenhagen, DK

204 Psychiatry, Indiana University School of Medicine, Indianapolis, IN, US

205 Biochemistry and Molecular Biology, Indiana University School of Medicine, Indianapolis, IN, US

# Supplementary tables

Supplementary table 1: SNPs with related genes associated with Alzheimer’s disease (AD) conditioned on their association with bipolar disorder (BIP) at a conditional false discovery rate <0.01.

| Locus no. | SNP | Closest gene | Chr | Position | P-value_(AD)_ | P-value_(BIP)_ | FDR_(AD)_ | condFDR_(AD\|BIP)_ |
| --- | --- | --- | --- | --- | --- | --- | --- | --- |
| 1 | rs6656401 | CR1 | 1 | 207692049 | 7.73E-15 | 5.04E-01 | 6.24E-06 | 8.35E-06 |
| 2 | rs6431219 | BIN1 | 2 | 127862133 | 7.63E-13 | 8.44E-01 | 6.24E-06 | 1.26E-05 |
| 2 | rs12617835 | LOC105373605 | 2 | 127886416 | 7.70E-13 | 3.23E-01 | 6.24E-06 | 5.89E-06 |
| 2 | rs56368748 | LOC105373605 | 2 | 127894098 | 7.16E-11 | 8.57E-01 | 7.25E-06 | 1.55E-05 |
| 3 | rs2878896 | HBEGF | 5 | 139710507 | 9.22E-08 | 9.76E-02 | 1.76E-03 | 2.25E-03 |
| 4 | rs77212406 | HLA-DRB5 | 6 | 32578209 | 6.84E-09 | 1.00E-01 | 2.57E-04 | 2.10E-04 |
| 5 | rs9381563 | CD2AP | 6 | 47432637 | 5.30E-09 | 5.86E-01 | 2.09E-04 | 5.45E-04 |
| 6 | rs11763230 | EPHA1-AS1 | 7 | 143108841 | 2.11E-11 | 4.47E-01 | 6.24E-06 | 7.60E-06 |
| 6 | rs12540656 | EPHA1-AS1 | 7 | 143117919 | 1.17E-08 | 3.46E-01 | 3.80E-04 | 7.72E-04 |
| 7 | rs28834970 | PTK2B | 8 | 27195121 | 3.27E-09 | 7.26E-01 | 1.38E-04 | 4.15E-04 |
| 8 | rs9331896 | CLU | 8 | 27467686 | 9.63E-17 | 2.48E-01 | 6.24E-06 | 4.79E-06 |
| 9 | rs71475924 | NDUFS3 | 11 | 47603006 | 1.44E-06 | 3.28E-03 | 1.50E-02 | 3.23E-03* |
| 10 | rs1530914 | MS4A4A | 11 | 60028940 | 5.60E-11 | 4.10E-01 | 6.24E-06 | 7.11E-06 |
| 11 | rs659023 | PICALM | 11 | 85824859 | 1.17E-12 | 5.03E-01 | 6.24E-06 | 8.34E-06 |
| 12 | rs11218343 | SORL1 | 11 | 121435587 | 4.98E-11 | 7.19E-01 | 6.24E-06 | 1.11E-05 |
| 13 | rs10143128 | RPS6KL1 | 14 | 75398902 | 1.00E-07 | 3.26E-01 | 1.88E-03 | 4.60E-03 |
| 14 | rs12590654 | SLC24A4 | 14 | 92938855 | 4.10E-08 | 2.16E-01 | 9.71E-04 | 1.72E-03 |
| 15 | rs12597717 | MTSS1L | 16 | 70702758 | 9.85E-06 | 1.25E-04 | 4.78E-02 | 5.57E-03* |
| 16 | rs8093731 | DSG2 | 18 | 29088958 | 4.63E-08 | 6.46E-01 | 1.06E-03 | 3.47E-03 |
| 17 | rs4147929 | ABCA7 | 19 | 1063443 | 1.70E-09 | 5.04E-01 | 7.68E-05 | 1.81E-04 |
| 18 | rs17878252 | FBXO46 | 19 | 46234155 | 7.87E-08 | 1.38E-01 | 1.56E-03 | 2.37E-03 |
| 19 | rs3865444 | CD33 | 19 | 51727962 | 5.12E-08 | 3.52E-01 | 1.14E-03 | 2.73E-03 |

* loci enriched for associations with BIP (condFDR_(AD|BIP)_ < FDR_(AD)_) not detected by conventional methods in original GWAS (p-value_(AD)_>5 x 10^-8^)

Supplementary table 2: SNPs with related genes associated with bipolar disorder (BIP) conditioned on their association with Alzheimer’s disease (AD) at a conditional false discovery rate <0.01.

| Locus no. | SNP | Closest gene | Chr | Position | P-value_(BIP)_ | P-value_(AD)_ | FDR_(BIP)_ | condFDR_(BIP\|AD)_ |
| --- | --- | --- | --- | --- | --- | --- | --- | --- |
| 1 | rs1889778 | LOC105378763 | 1 | 61066279 | 2.32E-07 | 5.49E-02 | 9.85E-03 | 7.91E-03* |
| 2 | rs57681866 | VRK2 | 2 | 57975714 | 5.00E-08 | 9.21E-02 | 5.81E-03 | 3.53E-03 |
| 3 | rs4619651 | LMAN2L | 2 | 97416153 | 5.97E-09 | 5.12E-01 | 2.30E-03 | 2.83E-03 |
| 4 | rs13011184 | CNTNAP5 | 2 | 125089268 | 9.26E-06 | 5.91E-04 | 3.73E-02 | 8.39E-03* |
| 5 | rs9834970 | TRANK1 | 3 | 36856030 | 5.53E-14 | 6.68E-01 | 4.28E-04 | 1.46E-05 |
| 6 | rs2071044 | ITIH4 | 3 | 52847601 | 9.10E-09 | 5.01E-01 | 2.74E-03 | 3.84E-03 |
| 7 | rs45605540 | KIAA1109 | 4 | 123141054 | 8.24E-07 | 1.18E-02 | 1.24E-02 | 7.38E-03* |
| 8 | rs7707981 | SSBP2 | 5 | 80922749 | 2.76E-07 | 1.42E-02 | 1.04E-02 | 3.37E-03* |
| 9 | rs329319 | JADE2 | 5 | 133906609 | 1.54E-08 | 6.48E-03 | 3.48E-03 | 1.32E-04 |
| 10 | rs55648125 | TFAP2B | 6 | 50816718 | 4.92E-08 | 3.15E-01 | 5.78E-03 | 8.84E-03 |
| 11 | rs2388334 | AK091365 | 6 | 98591622 | 8.62E-08 | 9.36E-02 | 6.99E-03 | 5.45E-03* |
| 12 | rs12672003 | MPP6 | 7 | 24647222 | 2.87E-08 | 5.72E-01 | 4.65E-03 | 9.74E-03 |
| 13 | rs12538191 | MYO1G | 7 | 44980824 | 1.46E-07 | 1.05E-01 | 8.36E-03 | 8.72E-03 |
| 14 | rs4980532 | RCOR2 | 11 | 63680719 | 8.95E-07 | 5.85E-04 | 1.26E-02 | 1.04E-03* |
| 15 | rs11237821 | TENM4 | 11 | 79106804 | 1.17E-08 | 6.75E-01 | 3.07E-03 | 6.01E-03 |
| 16 | rs10744560 | CACNA1C | 12 | 2387099 | 2.92E-09 | 3.40E-01 | 1.82E-03 | 1.09E-03 |
| 17 | rs4447398 | STARD9 | 15 | 42904904 | 1.10E-07 | 2.57E-02 | 7.57E-03 | 2.46E-03* |
| 18 | rs71395455 | ZSCAN2 | 15 | 85153804 | 1.93E-08 | 2.06E-01 | 3.88E-03 | 3.24E-03 |
| 19 | rs11647445 | GRIN2A | 16 | 9926966 | 1.22E-07 | 4.14E-02 | 7.84E-03 | 3.89E-03* |
| 20 | rs61554907 | THRA | 17 | 38220432 | 4.32E-06 | 3.33E-04 | 2.54E-02 | 4.33E-03* |
| 21 | rs876720 | HLF | 17 | 53366515 | 8.33E-09 | 4.57E-01 | 2.63E-03 | 3.31E-03 |
| 22 | rs7406066 | PRKCA | 17 | 64313153 | 6.25E-06 | 6.41E-04 | 3.05E-02 | 5.98E-03* |
| 23 | rs111444407 | NCAN | 19 | 19358207 | 2.40E-10 | 5.61E-01 | 1.24E-03 | 1.96E-04 |
| 24 | rs138321 | SLC25A17 | 22 | 41209304 | 4.69E-09 | 4.09E-01 | 2.11E-03 | 1.91E-03 |

* loci enriched for associations with AD (condFDR_(BIP|AD)_ < FDR_(BIP)_) not detected by conventional methods in original GWAS (p-value_(BIP)_>5 x 10^-8^)

# Supplementary figures


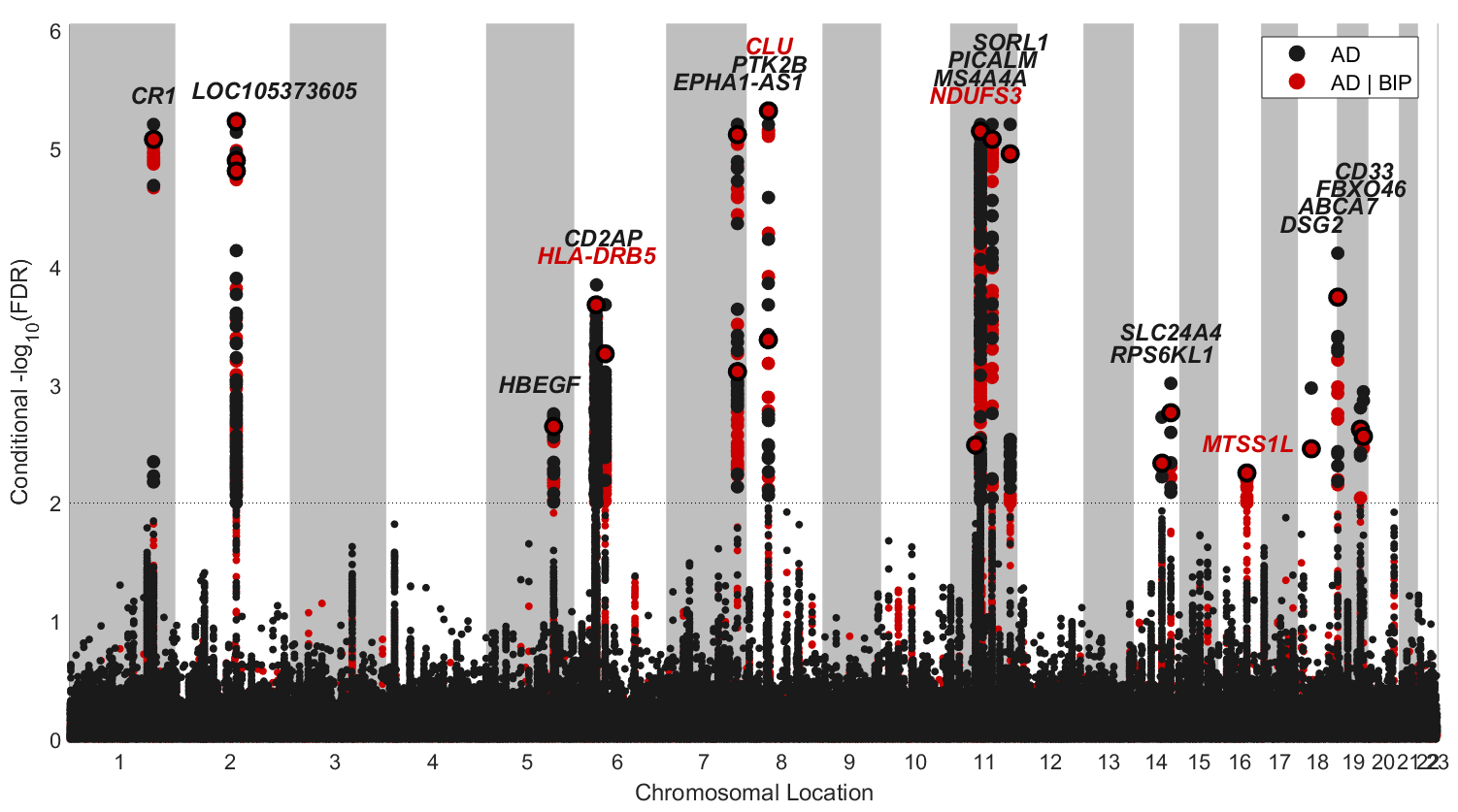


Supplementary figure 1: Conditional Manhattan plot of loci associated with Alzheimer’s disease at a conditional false discovery rate (condFDR) <0.01.


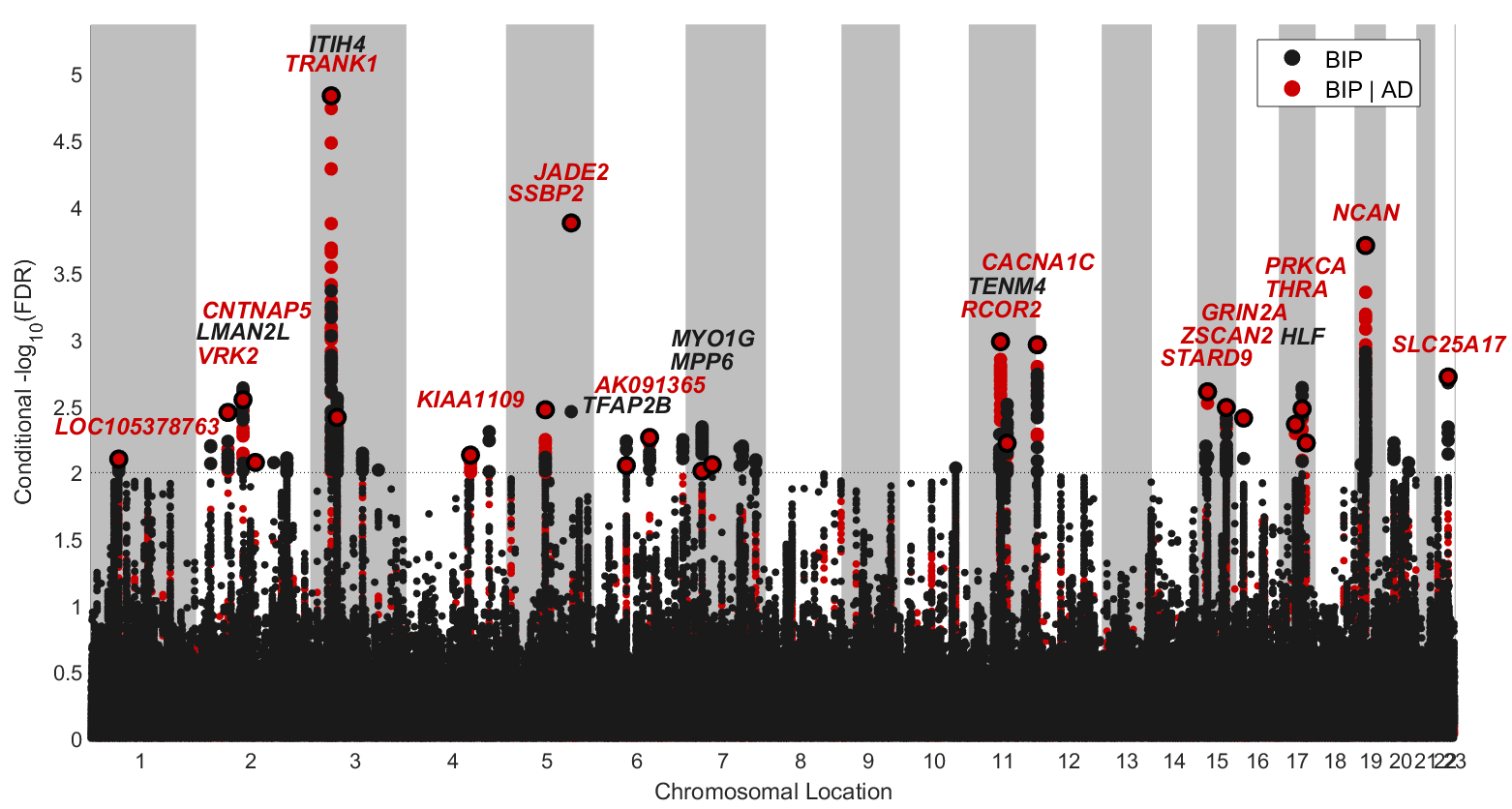


Supplementary figure 2: Conditional Manhattan plot of loci associated with bipolar disorder (BIP) at a conditional false discovery rate (condFDR) <0.01.

Supplementary figure 3: Expression of the MARK2 gene in different parts of the developing and adult human brain obtained from the Human Brain Transcriptome atlas^[[1]](#footnote-1)^ (Kang et al., 2011) (NCX neocortex, HIP hippocampus, AMY amygdala, STR striatum, MD mediodorsal nucleus of the thalamus, CBC cerebellar cortex).

Supplementary figure 4: Expression of the VAC14 gene in different parts of the developing and adult human brain obtained from the Human Brain Transcriptome atlas^[[2]](#footnote-2)^ (Kang et al., 2011) (NCX neocortex, HIP hippocampus, AMY amygdala, STR striatum, MD mediodorsal nucleus of the thalamus, CBC cerebellar cortex).

# Supplementary references

Andreassen, O. A., Desikan, R. S., Wang, Y., Thompson, W. K., Schork, A. J., Zuber, V., et al. (2015). Abundant genetic overlap between blood lipids and immune-mediated diseases indicates shared molecular genetic mechanisms. *PLoS One* 10. doi:10.1371/journal.pone.0123057.

Andreassen, O. A., Djurovic, S., Thompson, W. K., Schork, A. J., Kendler, K. S., O’Donovan, M. C., et al. (2013). Improved detection of common variants associated with schizophrenia by leveraging pleiotropy with cardiovascular-disease risk factors. *Am. J. Hum. Genet.* 92, 197–209.

Bulik-Sullivan, B., Finucane, H. K., Anttila, V., Gusev, A., Day, F. R., Loh, P.-R., et al. (2015a). An atlas of genetic correlations across human diseases and traits. *Nat. Genet.* 47, 1236–1241.

Bulik-Sullivan, B. K., Loh, P.-R., Finucane, H. K., Ripke, S., Yang, J., Patterson, N., et al. (2015b). LD Score regression distinguishes confounding from polygenicity in genome-wide association studies. *Nat. Genet.* 47, 291–295.

Kang, H. J., Kawasawa, Y. I., Cheng, F., Zhu, Y., Xu, X., Li, M., et al. (2011). Spatio-temporal transcriptome of the human brain. *Nature* 478, 483–489.

**
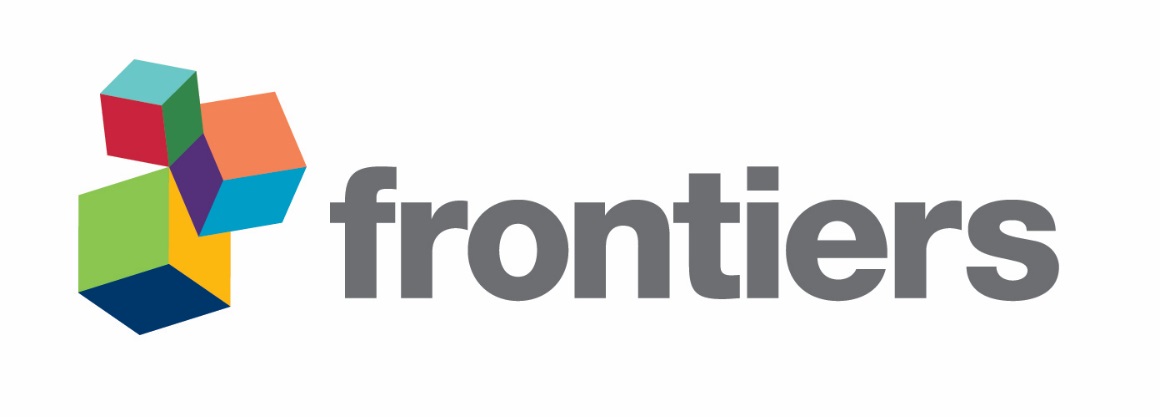
**

1. <http://hbatlas.org> [↑](#footnote-ref-1)
2. <http://hbatlas.org> [↑](#footnote-ref-2)
